# Effective Solutions for Caregivers of Older Adults: A Review of Systematic Reviews

**DOI:** 10.1177/07334648241312999

**Published:** 2025-01-09

**Authors:** Molly McHugh, Ellen Munsterman, Hannah Cho, Mary D. Naylor

**Affiliations:** 1 NewCourtland Center for Transitions and Health, 16142 University of Pennsylvania School of Nursing, Philadelphia, PA, USA; 2 Leonard Davis Institute for Health Economics, 6572 University of Pennsylvania, Philadelphia, PA, USA

**Keywords:** caregivers, older adults, biobehavioral interventions, gerontology, systematic review

## Abstract

This umbrella review aims to describe caregiver interventions tested across populations of informal caregivers of older adults and to examine the effect of caregiver interventions on depression, burden, and quality of life across intervention types and care-recipient populations. PubMed, Scopus, APA PsycInfo, ABIM, Sociological Abstracts, PAIS, and EconLit databases were systematically searched. Forty systematic reviews were identified as meeting inclusion criteria. Most commonly, interventions focused on improving outcomes for caregivers of older adults with dementia. Among the included reviews, caregiver depression was most likely to be reduced by caregiver interventions, and cognitive behavioral therapy (CBT) and mindfulness-based interventions (MBI) were most effective. The use of information and communication technologies to deliver caregiver interventions is increasingly common. Standardization of intervention classifications and transparent reports of intervention delivery details will strengthen research in this field.

**What this paper adds**

• Caregiver interventions demonstrated statistically significant improvements in depression, particularly among interventions focused on MBI and CBT.
• Systematic reviews of caregiver interventions have commonly focused on caregivers of older adults with dementia.
• Caregiver interventions continue to have different definitions and applications, limiting a broad synthesis of efficacy across intervention types.

**Applications of study findings**

• Researchers should increase descriptions of caregiver intervention design and components.
• Future research and reviews of caregiver interventions should explore interventions targeted at caregivers of older adults with non-dementia diagnoses.
• Caregiver interventions should increasingly integrate information and communication technologies.

## Background and Objectives

A growing number of older adults expect to require the support of caregivers in the future, but the number of paid caregivers is decreasing (National Association of Chronic Disease Directors, 2018). There are more than 56 million older adults living in the United States, and this population is projected to increase to 95 million by 2060 (Vespa et al., 2018). Additionally, over 6.5 million Americans are currently living with Alzheimer’s disease, just one type of cognitive impairment (Alzheimer’s Association, 2023). While the incidence of this disease is declining, the prevalence continues to grow alongside our rapidly aging population (Alzheimer’s Association, 2023). The growth in this population, coupled with increasing rates of chronic comorbidity and cognitive impairment, necessitates increasingly complex support for these individuals, provided largely by informal caregivers.

Caregivers of older adults face the compounding challenges of an aging population living longer with complex health and social needs, an expensive, overburdened, and fragmented healthcare system, and balancing caregiving with other responsibilities, including management of personal health, employment, and caring for children. The group of individuals who have and will increasingly bear the burden of caring for this older adult population is informal caregivers. For the purposes of this review, informal caregivers are defined as family members or friends, 18 years of age and older, who help individuals living with serious illnesses or disabilities to meet their physical, emotional, social, or financial needs, most often without payment for their services. Unfortunately, the U.S. care system has been largely ineffective in supporting this group. Interventions designed to meet the individualized needs of informal caregivers are urgently needed. Describing the effectiveness of existing interventions with sufficient evidence to recommend scaling is essential to better position the care system to respond to this crisis.

Among the most common measured negative effects are caregiver depression, burden, and decreased quality of life. High-priority outcomes, defined as concepts caregivers perceived as highly impactful to their wellbeing were selected as the primary outcomes for this review (Nadash et al., 2021; Novais et al., 2017). Informal caregivers experience significant differences in depression and burden compared to their non-caregiver counterparts, and this disparity further increases when examining caregivers of older adults living with persons living with dementia (Collins & Kishita, 2020; Pinquart & Sörensen, 2003). Informal caregivers also experience variable impacts on their health based on race and ethnicity, time spent caregiving, and the health of their care recipients (AARP & National Alliance for Caregiving, 2020; Parker et al., 2022; Riffin et al., 2017). A recent meta-analysis revealed that roughly half of caregivers of persons living with dementia report moderate to severe caregiver burden, and approximately a third of caregivers of persons living with dementia report depression (Collins & Kishita, 2020). Caregiving responsibilities can also have a considerable negative impact on caregivers’ quality of life, especially for caregivers of persons living with dementia (Moraes & Silva, 2009). For example, increased neuropsychiatric symptoms of care recipients living with dementia are associated with caregivers’ worsened mental health (Contreras et al., 2021). The wide-ranging impacts of caregiving on caregivers of older adults have resulted in extensive development of interventions to minimize the impact of caregiving.

A major challenge to scaling existing informal caregiver solutions is the highly variable nature of strategies targeting caregivers. Some interventions are targeted to a specific group of caregivers, thus limiting the generalizability of findings. Others are evaluated across a broad range of caregivers, but the identification of the specific types and components that are most effective is challenged due to substantial differences in the nature of these interventions. Recent umbrella reviews involving interventions of caregivers of older adults have focused on specific caregiver outcomes, such as the negative health outcomes to caregivers (Kirvalidze et al., 2023, or targeted particular caregiver populations, such as interventions focused on caregivers of individuals living with dementia (Naunton Morgan et al., 2022). This review expands upon previous caregiver research by delineating the types of interventions tested with diverse caregiver populations, emphasizing the use of technology in these interventions, and focusing on interventions that examine high-priority outcomes. Importantly, this approach will facilitate the identification of both promising solutions for large-scale spread and evaluation as well as critical gaps in research that need immediate attention. Thus, the aims of this umbrella review are to: (1) describe the intervention types tested across populations of caregivers of older adults, inclusive of interventions delivered via technology, and (2) examine the effect of caregiver interventions on depression, burden, and quality of life across intervention types and care-recipient populations.

## Research Design and Methods

This umbrella review was guided by the PRISMA Guidelines for Systematic Reviews and was registered through the Center of Open Science’s Open Science Framework (OSF) protocol registry (Foster & Deardorff, 2017; Page et al., 2021). Umbrella reviews use established methods to extract and analyze results across outcomes, using systematic reviews as the unit of analysis and allowing for research questions to address new questions not identified in the original reviews. Databases from diverse disciplinary perspectives, including PubMed, Scopus, APA PsycInfo, ABIM, Sociological Abstracts, PAIS, and EconLit, were systematically searched in 2023 to capture a broad sample of current caregiver interventions. The search included terms related to “caregiv*” and “older adult” (Table 1, Appendix A). The search was limited to peer-reviewed reviews published from January 2018 to January 2023 to provide a focus on contemporary caregiver interventions. Full eligibility criteria are described in Table 2. The search strategy was developed in collaboration with a University librarian.

**Table 1.** PICO Table.

Population	This study focuses on interventions targeting caregivers of older adults who are 65 years of age and older. As many systematic reviews on caregiver interventions include studies based on care recipients’ diagnoses instead of age, this study also includes reviews focused exclusively on age-related diagnoses. Age-related conditions included heart failure, Alzheimer’s or other related dementias, or chronic kidney disease.
Intervention	This study included reviews that exclusively evaluated the effects of interventions designed for caregivers of older adults and assessed via randomized control trials (RCTs).
Comparator	Comparators in the RCTs included control, control-waitlist, and usual care groups.
Outcomes	Studies measuring caregiver outcomes of caregiver depression, burden, and quality of life were eligible for inclusion in this review.

**Table 2.** Eligibility Criteria.

Eligibility Criteria	
Inclusion Criteria	Exclusion Criteria
Systematic reviews of randomized controlled trials (RCTs) evaluating interventions targeted exclusively at informal caregivers of older adults (65+) OR living with age-related conditions • If reported explicitly by the review, the average age of participants of included studies was 65 years or older • Age-related conditions included heart failure, Alzheimer’s disease or other related dementias, or chronic kidney disease (if the diagnosis was unclear, authors met to mutually agree on study eligibility)	Systematic reviews of RCTs evaluating interventions targeted as caregivers of adults (<65) or pediatric populations
Reviews reported on outcomes of the intervention for informal caregivers, including depression, burden and/or quality of life	Caregiver outcomes collected for care-recipient focused interventions
Published in the last 5 years (January 2018–January 2023)	Published before January 2018
Published in the English language	Not available in English
	Manuscripts that did not report on a form of knowledge synthesis (systematic review, scoping review, etc.)
	Reviews that included non-RCT studies

**Figure 1.**
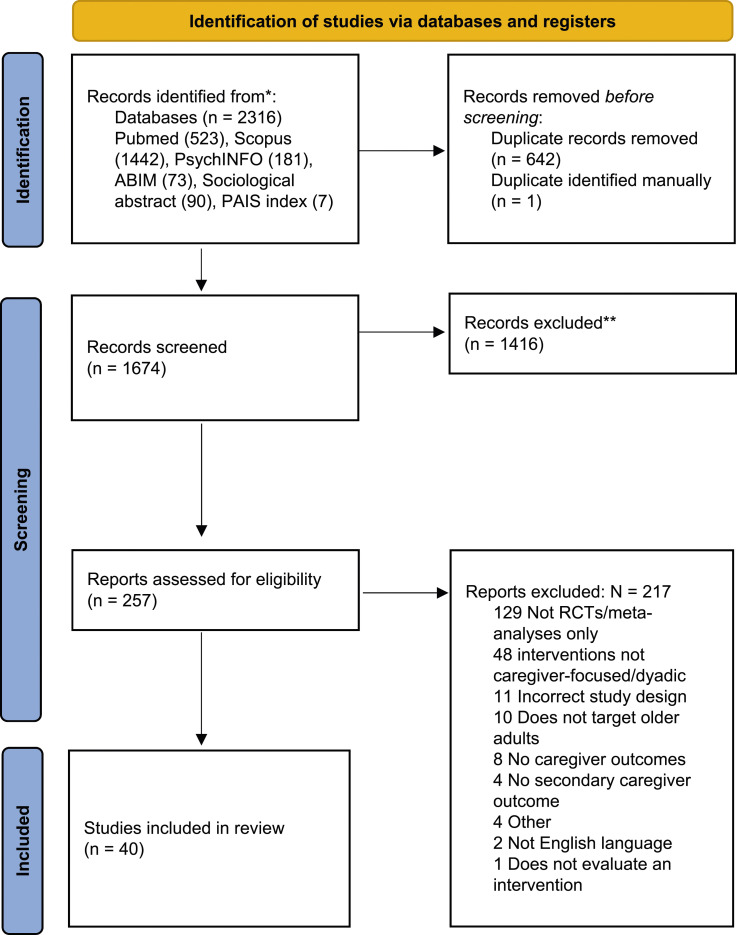
PRISMA flow diagram.

Search results were uploaded from databases to Covidence software for deduplication, abstract review, and data management (Covidence Systematic Review Software, 2024). Deduplication was first completed using Covidence, and then manually by authors. Two authors applied abstract review eligibility criteria and independently reviewed full texts (MM, EM). If there were disagreements between individual judgments, the reviewers met and successfully adjudicated all disagreements. Three authors abstracted (MM, HC, EM) data in an Excel table designed a priori that specified the intervention type and presence of technology component(s). Data on measured outcomes was formatted into a table of evidence compiled in Word, including author, year of publication, number of studies in the review, date range of the review, review objective, target population, intervention type(s), and reported biases/limitations. Results of abstraction were reviewed by three authors (MM, HC, and EM), and any discrepancies were discussed and reconciled. The PRISMA Flowchart describes this review’s study identification process (Figure 1).

Due to the heterogeneity of caregiver interventions, researchers categorized intervention types to compare their efficacy with those with similar components. Such classification is an essential element in reviewing caregiver interventions across diverse populations and components. Gaugler and colleagues have classified caregiver interventions within the dementia literature, calling attention to the importance of such categorization to allow for more precise efficacy assessments (Gaugler et al., 2017). Our review applied Cheng and colleagues’ nine categories of caregiver interventions, which expanded Gaugler and colleagues’ classifications by including a miscellaneous intervention type to capture novel interventions that do not fall into existing categories (Cheng et al., 2020, 2022) (Table 3).

**Table 3.** Intervention Types and Definitions.

Intervention Types	
Psychoeducation	Psychoeducational interventions include educational programs with probable psychological components to improve coping and/or psychotherapeutic components. These interventions may include developing specific coping skills to deal with challenges in caregiving and group psychotherapy.
Counseling & psychotherapy	Counseling & psychotherapy interventions involve the implementation of specified forms of individual or group therapy or counseling. They are distinguished from psychoeducational programs in that they usually use professional psychologists or therapists as interventionists and place stronger emphasis on the development and utilization of the therapeutic relationship as part of the treatment process. Studies have usually used behavior therapy, cognitive therapy, and conventional CBT but newer theoretical orientations such as acceptance-commitment therapy are also included.
Training of the CR with caregiver involvement	These interventions involving direct training of the CR (e.g., reminiscence, cognitive stimulation, occupational therapy, exercise) while involving the caregiver as an integral part of the intervention (e.g., administering designated activity, providing direct supervision, or devising activities tailor-made for the CR.
Multicomponent	Multicomponent interventions include multiple intervention approaches, such as counseling, support groups, and respite, without any one being the dominating component. Programs that provide one type of intervention to the caregiver and another type to the CR simultaneously are also considered multicomponent interventions.
Mindfulness-based interventions	Mindfulness-based interventions include interventions focused on mindfulness, meditation, and yoga techniques in helping caregivers. Although they are not synonymous with each other, these interventions operate on similar principles and are grouped together. Note that mindfulness-based cognitive therapy is also considered to be a form of psychotherapy but is grouped under this category because of its similarity to other mindfulness approaches such as mindfulness-based stress reduction.
Support groups	These occur when arrangements are made to connect caregivers with one another so that they can provide support to each other in groups, which may or may not be facilitated by program staff.
Respite/Day care	This category of interventions involves the provision of short-term breaks from caregiving. With a goal of promoting caregiver rest and reducing caregiver stress, these interventions may involve in-home care and adult day care centers.
Care coordination & case management	Health care or allied health professionals act to increase access to services, match services to caregiver/CR preferences and needs, and coordinate services to avoid duplication and unnecessary utilizations. Unlike the above, the emphasis is not on the introduction of a direct intervention program but on the improvement of existing service delivery.
Miscellaneous	This category consists of a range of programs that cannot be readily classified into the above categories and which cannot form stand-alone categories themselves because of the small number of studies. Examples are physical exercise for caregivers, communication training, spiritual care, decision-making training, and various complementary/alternative therapies.

Adapted from Cheng et al. (2020).

### Quality Assessment

Review quality was assessed using “A Measurement Tool to Assess Systematic Reviews (AMSTAR) 2” (Shea et al., 2017). This is an appropriate screening tool, as it evaluates biases of systematic reviews (Shea et al., 2017). Three authors (MM, EM, and HC) independently applied AMSTAR 2 criteria to each of the included reviews. AMSTAR 2 recommends that this appraisal tool be tailored to research aims, and that an overall score is not recommended (Shea et al., 2017). The authors jointly reviewed all scoring to identify essential questions tailored to the aims of the paper and met for further discussion only if there were disagreements in scoring. Disagreements in scoring were uncommon across AMSTAR 2 questions. Questions 2, 4, 9, 11, 13, and 15 were identified as essential to the AMSTAR 2 interpretation of the study and a “No” response to these questions was marked as a critical flaw. All other questions with “No” responses were considered non-critical weaknesses.

### Synthesis

We applied narrative data synthesis for our umbrella review, organizing information by intervention type, diagnosis, and primary caregiver outcomes measured. Narrative synthesis is an appropriate umbrella review synthesis method to analyze diverse qualitative and quantitative information across disciplines (Aromataris et al., 2014). The systematic reviews (referred to as “reviews” throughout this paper) included in our umbrella review summarized studies both qualitatively using narrative syntheses, as well as quantitatively, including analytic approaches like meta-analyses. We synthesized caregiver outcomes *within* and *across* reviews of similar intervention types and care recipient diagnoses in order to narratively describe associations between similar reviews; this approach is a central element of effective narrative synthesis as described by the Cochrane Manual for Interventions (Higgins & Green, 2008). When meta-analyses were conducted, we reported the effect size using mean difference or standardized mean difference when available, as well as measures of heterogeneity (Appendix B).

## Results

### Overview of Included Reviews

Of the 257 reports assessed for eligibility, this search identified 40 reviews that met inclusion criteria and assessed caregiver depression, burden, and quality of life as outcomes (Figure 1). Appendix B provides a table of evidence for the 40 included reviews. Review findings were reported based on review type and outcome. For included systematic reviews, a narrative summary of studies identifying target outcomes are summarized. For included meta-analyses, the significance, directionality, and effect of each target outcome are reported. Effect size was commonly reported using standard mean difference (SMD), but effect sizes are listed using the original statistical methods of the review authors.

As study authors use varying definitions of intervention types across reviews, we listed the types of interventions based on an application of Cheng and colleagues’ definitions of caregiver intervention categories in order to more clearly compare outcomes across intervention types (Cheng et al., 2020, 2022) (Table 3). Using these criteria, reviews were categorized by the aims of the review (i.e., intervention-driven, diagnosis-driven, or a combination of both). We operationally defined diagnosis-driven as reviews that targeted interventions on specific health conditions, and intervention-driven as reviews that targeted specific intervention types in the inclusion and exclusion criteria. Among 40 reviews, 3 reviews were intervention-driven, 12 were diagnosis-driven, and 25 were both intervention and diagnosis-driven. Ten of the reviews included in our umbrella review were appraised as “Critically Low,” 11 were appraised as “Low,” 19 were appraised as “Moderate,” and one was appraised as “High” quality (Appendix C). There were several AMSTAR 2 questions where most of the reviews received a “No” response. These responses were primarily due to the lack of providing (1) a rationale for study design inclusion, (2) a list of excluded articles and justifying exclusions, and (3) a list of funding sources. Individual reviews were not removed from our umbrella review based on the results of the quality appraisal because the issues were largely related to how the systematic review was reported, not with the individual studies included in each review.

### Intervention Types

Cheng and colleagues’ definitions of caregiver intervention categories were applied to identify which intervention types were described most frequently within included reviews. Of the 40 included reviews, 28 reviews examined the effects of psychoeducational approaches, the most common type of intervention (Akarsu et al., 2019; Andrades-González et al., 2021; Chacko et al., 2022; Cheng et al., 2020; Cheng et al., 2022; Chin et al., 2022; Egan et al., 2018; Frambes et al., 2018; Frias et al., 2020; Kishita et al., 2018; Knowles et al., 2021; Lee, Ryoo, Chung, et al., 2020; Lee, Ryoo, Crowder, et al., 2020; Lee et al., 2021; Liu et al., 2022; Lucero et al., 2019; Mårtensson et al., 2023; Moore et al., 2020; Mou et al., 2021; Sun, Ji, Leng, & Wang, 2022; Teahan et al., 2020; Wang et al., 2021; Wiegelmann et al., 2021; Williams et al., 2019; Zhao et al., 2019; Zheng et al., 2021; Zhu et al., 2021; Zulkifley et al., 2020). Multicomponent interventions were the second most common type of intervention identified in this study (*n* = 26) (Agustina et al., 2023; Akarsu et al., 2019; Chacko et al., 2022; Cheng et al., 2020; Cheng et al., 2022; Frias et al., 2020; Han, 2022; He et al., 2022; Hovadick et al., 2021; Lee, Ryoo, Chung, et al., 2020; Lee et al., 2021; Liu et al., 2022; Lucero et al., 2019; Mårtensson et al., 2023; Moore et al., 2020; Mou et al., 2021; Sun, Ji, Leng, & Wang, 2022; Teahan et al., 2020; Wang et al., 2020; Wang et al., 2021; Wiegelmann et al., 2021; Williams et al., 2019; Zabihi et al., 2020; Zhao et al., 2019; Zheng et al., 2021; Zulkifley et al., 2020). Twenty-three reviews examined the effects of counseling and psychotherapy (Akarsu et al., 2019; Andrades-González et al., 2021; Chacko et al., 2022; Cheng et al., 2020, 2022; Frambes et al., 2018; Han, 2022; Kishita et al., 2018; Lee et al., 2020a, 2020b, 2021; Liu et al., 2022; Lucero et al., 2019; Mårtensson et al., 2023; Sun et al., 2022a, 2022b; Teahan et al., 2020; Wang et al., 2021; Wiegelmann et al., 2021; Williams et al., 2019; Zabihi et al., 2020; Zheng et al., 2021; Zhou et al., 2022; Zhu et al., 2021). Seventeen reviews evaluated interventions of the miscellaneous type (Agustina et al., 2023; Baik et al., 2021; Cheng et al., 2020; Knowles et al., 2021; Kustanti et al., 2021; Lee et al., 2020a, 2021; Lucero et al., 2019; Martis et al., 2022; Sun, Ji, Leng, & Wang, 2022; Teahan et al., 2020; Wang et al., 2020, 2021; Wiegelmann et al., 2021; Williams et al., 2019; Zheng et al., 2021). Support groups were examined in twelve reviews of interventions (Andrades-González et al., 2021; Cheng et al., 2020, 2022; Hovadick et al., 2021; Lee et al., 2020a, 2020b, 2021; Lucero et al., 2019; Sun, Ji, Leng, & Wang, 2022; Wang et al., 2021; Zheng et al., 2021; Zhu et al., 2021). Nine reviews examined mindfulness-based interventions (Baik et al., 2021; Chacko et al., 2022; Cheng et al., 2020, 2022; Han, 2022; Lee, Ryoo, Chung, et al., 2020; Martis et al., 2022; Wang et al., 2021; Zheng et al., 2021). Five reviews examined the effects of care coordination and case management interventions (Cheng et al., 2022; Lee, Ryoo, Chung, et al., 2020; Lucero et al., 2019; Sun, Ji, Leng, & Wang, 2022; Wang et al., 2020). Finally, two reviews discussed training interventions involving both the care recipient and caregivers (Bennett et al., 2019; Frambes et al., 2018). As many of the included reviews focused on a specific outcome or diagnosis type, it was common for a single review to include numerous intervention types, thus the recurrence of individual reviews in intervention categories.

### Caregiver Outcomes

This umbrella review identified reviews that evaluated caregiver depression, caregiver quality of life, or caregiver burden. Meta-analytic findings from included reviews are compared by caregiver outcomes and are described narratively if no meta-analysis was completed.

#### Caregiver Depression

Of the 40 reviews, all but the following three assessed caregiver depression (Hovadick et al., 2021; Lee, Ryoo, Chung, et al., 2020; Williams et al., 2019). Eleven reviews measured caregiver depression outcomes narratively (Agustina et al., 2023; Baik et al., 2021; Chacko et al., 2022; Egan et al., 2018; Frambes et al., 2018; Frias et al., 2020; Lucero et al., 2019; Wang et al., 2021; Wiegelmann et al., 2021; Zheng et al., 2021; Zulkifley et al., 2020), while the remaining 26 reviews used meta-analytic methods to evaluate the impact of caregiver interventions. For those reviews describing caregiver depression outcomes narratively, caregiver interventions demonstrated mixed impacts on caregiver depression. Of the **26** reviews using meta-analytic methods, 23 of the reviews found caregiver interventions demonstrated statistically significant reductions in caregiver depression, and three found no or nonsignificant changes (Bennett et al., 2019; Knowles et al., 2021; Mou et al., 2021). Intervention types that demonstrated moderate to large effect sizes included mindfulness-based interventions (MBI) (Han, 2022; Lee, Ryoo, Chung, et al., 2020; Zabihi et al., 2020), CBT (Kishita et al., 2018; Lee, Ryoo, Chung, et al., 2020; Teahan et al., 2020), and psychosocial intervention types (Lee et al., 2021). One review also demonstrated a moderate effect of video and web-based bibliotherapy versus control (Wang et al., 2020), while another demonstrated a large effect for eHealth interventions versus control (Andrades-González et al., 2021).

#### Caregiver Burden

Twenty-seven reviews assessed the effects of interventions on caregiver burden, with 11 of these describing narrative findings (Agustina et al., 2023; Baik et al., 2021; Chacko et al., 2022; Frambes et al., 2018; Frias et al., 2020; Hovadick et al., 2021; Lucero et al., 2019; Wang et al., 2021; Wiegelmann et al., 2021; Zheng et al., 2021; Zulkifley et al., 2020). Among these reviews, caregiver interventions demonstrated mixed impacts on caregiver burden. Of the 16 reviews using meta-analytic methods, nine reviews found that caregiver interventions demonstrated statistically significant reductions in caregiver burden (Andrades-González et al., 2021; Cheng et al., 2020; He et al., 2022; Mårtensson et al., 2023; Martis et al., 2022; Mou et al., 2021; Sun, Ji, Leng, & Wang, 2022; Teahan et al., 2020; Williams et al., 2019), and seven found no or nonsignificant changes. For those reviews demonstrating statistically significant reductions in caregiver burden, effect sizes ranged from small to large, with the majority of reviews demonstrating a small effect size. One review demonstrated a statistically significant reduction in caregiver burden of yoga therapy versus control groups with a large effect size (Martis et al., 2022). Another review demonstrated a large effect for case management interventions versus control (Sun, Ji, Leng, & Wang, 2022).

#### Caregiver Quality of Life

Twenty-five reviews measured the impact of interventions on caregivers’ quality of life (QoL). Of these, eight reviews narratively evaluated caregiver QoL outcomes, with caregiver interventions demonstrating mixed impacts on caregiver quality of life (Baik et al., 2021; Egan et al., 2018; Frambes et al., 2018; Frias et al., 2020; Hovadick et al., 2021; Wang et al., 2021; Wiegelmann et al., 2021; Zheng et al., 2021). Of the 17 reviews using meta-analytic methods, eight found that caregiver interventions demonstrated statistically significant improvements in caregiver quality of life (Andrades-González et al., 2021; Bennett et al., 2019; Cheng et al., 2022; Han, 2022; He et al., 2022; Lee et al., 2020b, 2021; Sun, Ji, Leng, & Wang, 2022), and nine found no or nonsignificant changes (Chin et al., 2022; Kishita et al., 2018; Knowles et al., 2021; Martis et al., 2022; Mou et al., 2021; Teahan et al., 2020; Zhao et al., 2019; Zhou et al., 2022; Zhu et al., 2021). For those reviews demonstrating statistically significant improvements in caregiver quality of life, effect sizes ranged from small to large effect.

### Caregiver Outcomes by Diagnosis Type

Of the 33 reviews that specified a diagnosis type in the inclusion criteria, the three most common diagnoses were dementia, cancer, and stroke, described below. The remaining reviews focused on caregivers of care recipients with a variety of diagnoses, including end-stage renal disease (Hovadick et al., 2021), heart disease (Knowles et al., 2021), “dementia, MCI, stroke, and mixed diagnoses (Mårtensson et al., 2023), “chronic diseases/conditions” (Baik et al., 2021), individuals who are “terminally ill” (Zheng et al., 2021), or individuals receiving palliative care (Kustanti et al., 2021). Only one review focused on articles that looked specifically at caregiver characteristics, namely ethnic minority caregivers for persons living with dementia (Akarsu et al., 2019).

#### Dementia

Twenty-six reviews summarized findings of interventions for caregivers of persons with Alzheimer’s disease and related disorders. In sixteen of the twenty-six reviews describing caregivers of older adults living with dementia, statistically significant meta-analytic improvements on depression were demonstrated, (Akarsu et al., 2019; Cheng et al., 2020, 2022; Han, 2022; He et al., 2022; Kishita et al., 2018; Lee, Ryoo, Chung, et al., 2020; Liu et al., 2022; Martis et al., 2022; Moore et al., 2020; Sun et al., 2022a, 2022b; Teahan et al., 2020; Zabihi et al., 2020; Zhao et al., 2019; Zhu et al., 2021) while only one review demonstrated a nonsignificant meta-analytic effect on depression (Bennett et al., 2019). Seven reviews examined the effect of caregiver depression among caregivers of older adults living with dementia narratively, demonstrating promising but mixed reviews (Chacko et al., 2022; Egan et al., 2018; Frias et al., 2020; Lucero et al., 2019; Wang et al., 2021; Wiegelmann et al., 2021; Zulkifley et al., 2020). Han and colleagues reported a statistically significant reduction in depressive symptoms with moderate effect for MBIs versus control group, while Zabihi and colleagues found statistically significant reductions in depressive symptoms with a moderate to large effect pre- and post-behavioral activation interventions (Han, 2022; Zabihi et al., 2020). Kishita and colleagues reported a nonsignificant reduction in depression with small effect for psychoeducation-skill building interventions versus control group, but a statistically significant, moderate effect on depression reduction for CBT-based psychotherapeutic interventions (2018). Finally, Sun and colleagues found a large effect for CBT interventions via three delivery formats versus control group: internet, telephone, and individual (2022).

Six of the seventeen reviews assessing caregiver burden among caregivers of older adults with dementia reported statistically significant improvements in caregiver burden, (Cheng et al., 2020; He et al., 2022; Martis et al., 2022; Sun, Ji, Leng, & Wang, 2022; Teahan et al., 2020; Williams et al., 2019) while five reported nonsignificant effects on caregiver burden (Bennett et al., 2019; Kishita et al., 2018; Moore et al., 2020; Zhao et al., 2019; Zhu et al., 2021). Of those reporting statistically significant reductions in caregiver burden, the majority of reviews demonstrated small effects, while Martis and colleagues found a statistically significant reduction in burden with large effect for yoga interventions versus control groups (2023).

Finally, fifteen of the twenty-six reviews describing caregivers of older adults living with dementia reported on caregiver quality of life. Of these fifteen, six reviews reported statistically significant improvements in caregiver quality of life, (Bennett et al., 2019; Cheng et al., 2022; Han, 2022; He et al., 2022; Lee, Ryoo, Crowder, et al., 2020; Sun, Ji, Leng, & Wang, 2022) and five reviews reported nonsignificant meta-analytic effects (Kishita et al., 2018; Martis et al., 2022; Teahan et al., 2020; Zhao et al., 2019; Zhu et al., 2021). While the majority of reviews found small effects, Han and colleagues reported a statistically significant improvement in QOL with a large effect for MBIs versus control group at the immediate posttest (2022).

#### Cancer

Four reviews examined interventions that targeted caregivers of older adults living with cancer (Agustina et al., 2023; Frambes et al., 2018; Lee et al., 2021; Zhou et al., 2022). Of these, two reported statistically significant meta-analytic improvements in caregiver depression (Lee et al., 2021; Zhou et al., 2022). Lee et al. found a statistically significant reduction in depression with moderate effect in psychosocial interventions versus control group immediately post-intervention (2021). Zhou et al. reported statistically significant reductions in depression scores with a small effect for the CBT intervention versus control group (2022).

#### Stroke

Three papers reviewed interventions among caregivers of older adults with stroke (Andrades-González et al., 2021; Chin et al., 2022; Mou et al., 2021). Among these, all three assessed caregiver depression, with two of the three demonstrating a significant improvement on caregiver depression (Andrades-González et al., 2021; Chin et al., 2022). Chin and colleagues found a statistically significant reduction in depressive symptoms with a small effect for technology-based interventions group versus control group (2022). All three also assessed caregiver burden, with two of the three demonstrating a significant improvement on caregiver burden (Andrades-González et al., 2021; Mou et al., 2021). Only one of the three saw a significant increase in caregiver quality of life, while the remaining two reviews showed nonsignificant improvements in caregiver quality of life (Andrades-González et al., 2021).

### Caregiver Outcomes by Intervention Type

Reviews that specified an intervention type in their inclusion criteria and met Cheng and colleagues’ definitions of caregiver intervention categories were reviewed for outcomes by intervention type. Of these, the most commonly reviewed intervention categories were Psychoeducational, followed by Counseling and Psychotherapy, and Miscellaneous.

#### Psychoeducational Interventions

Six reviews were Psychoeducational (Chin et al., 2022; Egan et al., 2018; Frias et al., 2020; Moore et al., 2020; Mou et al., 2021; Zulkifley et al., 2020). Of the reviews focused on Psychoeducational interventions, three described depression outcomes narratively with mixed findings (Egan et al., 2018; Frias et al., 2020; Zulkifley et al., 2020). Another two reviews reported statistically significant meta-analytic improvements in caregiver depression (Chin et al., 2022; Moore et al., 2020), and one reported nonsignificant meta-analytic effects on caregiver depression (Mou et al., 2021). One review found that technology-based interventions with structured educational programs significantly reduced depressive symptoms (Chin et al., 2022).

#### Counseling and Psychotherapy Interventions

Three reviews focused on Counseling & Psychotherapy (Sun, Ji, Leng, Li, et al., 2022; Zabihi et al., 2020; Zhou et al., 2021). Each demonstrated statistically significant meta-analytic improvements in caregiver depression, and Zhou and colleagues reported nonsignificant meta-analytic effects on caregiver quality of life (2021). Two of these three reviews examined CBT therapies, demonstrating statistically significant reductions in caregiver depression, with small to large effects (Sun, Ji, Leng, Li, et al., 2022; Zhou et al., 2022).

#### Miscellaneous Interventions

Three reviews the examined Miscellaneous intervention type (Kustanti et al., 2021; Wang et al., 2020), two of which reported statistically significant meta-analytic improvements in caregiver depression (Kustanti et al., 2021; Wang et al., 2020).

### Technology

Delivering caregiver interventions using forms of information and communication technologies (ICT) is increasingly common. ICT includes delivery formats such as telephone calls, videoconferencing, web-based platforms, text messages, and other forms of electronic or digital communication. Of the 40 articles included in this review, only two reviews included studies where interventions were delivered exclusively via face-to-face without technology (Bennett et al., 2019; Hovadick et al., 2021), and in two reviews, insufficient detail was provided regarding the delivery method of the individual interventions to determine ICT use (Kustanti et al., 2021; Martis et al., 2022). The remaining 36 reviews included at least one study with an identified ICT delivery format. ICT delivery formats reported in the studies within our included reviews include telephone calls, videoconferencing, teleconferencing, video-phone support, web-based support, online platforms, text messaging, and materials delivered via DVD or CD-ROM. It was common for individual studies to initially deliver an intervention in person but then for follow-up sessions and/or further support to be provided using ICT. In some cases, the intervention group would receive their intervention face-to-face, while the comparison or control group would receive their intervention via ICT.

Six of the 40 included reviews had as a primary objective to evaluate the impact of an ICT delivery method of a caregiver intervention (Andrades-Gonzales et al., 2021; Chin et al., 2022; Egan et al., 2018; Lucero et al., 2019; Zhao et al., 2019; Zhu et al., 2021). Across the six **reviews** that evaluated exclusively ICT-based interventions, the outcome of caregiver depression was most consistently associated with significant results. Just one of the included reviews aimed to compare different types of delivery formats, including ICT (Sun, Ji, Leng, Li, et al., 2022). Sun and colleagues (2022b) used a network analysis to compare the delivery method of CBT for depression for caregivers of older adults with dementia. They compared internet, telephone, individual, group, and combined approaches for intervention delivery and determined that internet, telephone, and individual methods significantly reduced caregiver depression (Sun, Ji, Leng, Li, et al., 2022). Several authors included a discussion of the delivery format, including the use of ICT, even if formal comparisons were not part of the review (Akarusu et al., 2019; Baik et al., 2021; Hovadick et al., 2021; Wiegelmann et al., 2021; Zulkifley et al., 2020). Benefits of ICT delivery include convenience, addressing the challenge that caregivers face leaving the home, potential cost savings, and improved monitoring (especially with the use of wearable sensors) (Akarusu et al., 2019; Baik et al., 2021; Zulkifley et al., 2020). Challenges include cost, the delivery and installation of technological devices, equity issues related to the availability of wireless internet and personal devices, and that some methods require self-motivation and self-direction, which may be challenging for caregivers who are already burdened (Akarusu et al., 2019; Chacko et al., 2022; Zulkifley et al., 2020). Several studies included recommendations to assess digital literacy and consider the age of the potential user when considering an ICT-based delivery format for caregiving interventions.

## Discussion

This review of solutions for caregivers of older adults extends existing research by applying a structured categorization approach to describe the effects of rigorously tested interventions designed for caregivers who support older adults with diverse diagnoses on key caregiver outcomes of depression, burden, and quality of life. The main goal of this review was to identify interventions with sufficient evidence to warrant larger-scale spread and evaluation and to identify critical research gaps. We found that the vast majority of systematic reviews of caregiver interventions are focused on caregivers of older adults with dementia. Across diagnoses, however, the majority of meta-analytic reviews evaluating depression outcomes have demonstrated statistically significant reductions. Particularly promising among these are interventions focused on CBT and MBI. Among those reviews evaluating caregiver burden, findings were mixed, but the largest significant effects were found in a review described by Martis and colleagues (2022) evaluating yoga interventions (Martis et al., 2022). Reviews exploring caregiver quality of life also demonstrated mixed effects, with the largest effect, found that highly individualized caregiver interventions are most effective, especially among caregiver depression outcomes (Han, 2022; Kishita et al., 2018; Sun, Ji, Leng, Li, et al., 2022). Interventions involving CBT and MBI techniques are highly focused on the individual caregiver, often using professional psychologists or therapists to support a therapeutic interaction with caregivers (Han, 2022). Other caregiver reviews have also corroborated these findings, suggesting that multicomponent interventions that can be tailored to individual caregiver needs are effective in reducing negative health outcomes associated with caregiving (Kirvalidze et al., 2023). To support interventions that are further tailored to caregiver needs, findings from this umbrella review suggest that caregiver needs assessments may be an integral baseline component of caregiver interventions, providing nuanced insight into individualized caregivers’ needs.

This umbrella review also highlights how the intervention delivery method varied across studies, especially at the systematic review level, so making comparisons regarding intervention delivery can be difficult. The use of technology to deliver aspects of the intervention is growing, but this review suggests mixed evidence about the effectiveness of this delivery mode, which is further complicated by the wide variety of intervention categories and diagnosis types included in each review. However, this umbrella review does show that the involvement of multiple technologies in the administration of caregiver interventions can be appropriate and effective for caregivers of older adults.

The finding that the involvement of multiple technologies in caregiver interventions is appropriate for caregivers of older adults across diagnoses provides insight into the large-scale implementation of caregiver interventions. In order to implement professionally led, individualized interventions for caregivers of older adults at scale, technology may be helpful in providing therapeutic interactions with professionals in cases where time or transportation are limited. Technology facilitates interactions between healthcare professionals and caregivers, both minimizing transportation burdens for caregivers and expanding the reach of healthcare professionals. However, as addressed by Wiegelmann and colleagues, interventions must carefully consider the barriers to technology implementation as interventions are scaled (Wiegelmann et al., 2021). Involving multi-disciplinary teams, including technology professionals, will be critical as caregiver interventions grow.

This review identified that overall, systematic reviews of caregiver interventions across populations of caregivers of older adults with non-dementia diagnoses are limited. Future research and systematic reviews of caregiver interventions should explore interventions targeted at caregivers of older adults with non-dementia diagnoses. Furthermore, as recognized by other reviews of caregiver interventions, the varied categorizations of caregiver interventions continue to complicate a generalized interpretation of caregiver intervention efficacy (Egan et al., 2018). While this review applied a recognized intervention categorization structure, the individual reviews included in this study did not necessarily apply the same definitions and categorizations. To continue high-level analyses of caregiver interventions, future caregiver intervention research should provide detailed descriptions of intervention categories and components so that intervention efficacy can be reliably associated with intervention categories. As future caregiver interventions are developed and scaled, the application of recognized definitions and categorizations of interventions is essential to continue to interpret the efficacy of these caregiver solutions across expanded caregiver populations.

The reviews included in this umbrella review have certain limitations that should be considered when evaluating the overall findings. Firstly, as addressed in Appendix B, the use of various tools to measure multiple outcome variables in primary studies introduces variability and poses challenges in comparing the results. We applied a broad definition to caregivers in this review to account for the diverse group of individuals that care for older adults. However, it is possible that interventions targeting spouses or other family members did not use the terms caregiving or caregiver and thus were missed in our search. Additionally, the relatively small sample sizes and short intervention durations of the selected studies further limit researchers’ applicability and emphasize the need for future research. Not every study conducted a meta-analysis which hinders a comprehensive quantitative understanding of the overall effects. Finally, given the significant variability and heterogeneity in intervention contents, modes of delivery, and outcome measures, combined with the low methodological quality observed in this review, future intervention research must prioritize high-quality caregiver intervention research that extends existing knowledge and refines our understanding of caregiver interventions using detailed and rigorous methods.

## Conclusion

In this umbrella review, we synthesized the results of 40 systematic reviews and meta-analyses evaluating interventions for caregivers of older adults based on intervention type and care recipient diagnosis. The heterogeneous intervention types and delivery methods made comparisons challenging, but some patterns were clear and allowed for some broad conclusions and recommendations. Research to address outcomes of interventions for caregivers of older adults with dementia significantly outweighs all other care recipient diagnoses. Interventions to address caregivers of individuals with other diagnoses and with co-occurring cognitive impairment and other chronic illnesses are needed. CBT and mindfulness-based interventions, including yoga, had the best evidence across our included reviews, and among the three targeted outcomes, depression was most likely to be significantly reduced. Consistent with other reviews, we also identified that interventions tailored to the specific caregiver are most effective. Future testing of caregiver interventions should include needs assessments, adhere to standardized intervention definitions, and include detailed accounts of delivery methods, including the use of ICT, to allow for a better understanding of the most effective methods for addressing the needs of caregivers.

## Supplemental Material

Supplemental Material - Effective Solutions for Caregivers of Older Adults: A Review of Systematic Reviews Supplemental Material for Effective Solutions for Caregivers of Older Adults: A Review of Systematic Reviews by Molly McHugh, Ellen Munsterman, Hannah Cho, and Mary D. Naylor in Journal of Applied Gerontology
