# Supplementary material for: Effective Solutions for Caregivers of Older Adults: A Review of Systematic Reviews
Source: J Appl Gerontol. 2025 Jan 9;44(10):1571–83. doi: 10.1177/07334648241312999 (PMC12238300; doi:10.1177/07334648241312999)
Supplement: Supplemental Material - Effective Solutions for Caregivers of Older Adults: A Review of Systematic Reviews [file sj-pdf-1-jag-10.1177_07334648241312999.pdf]

Appendix A: Search Terms

Database	Date Searched	Search Terms
ABIM/Inform	1/31/23	(MAINSUBJECT.EXACT("Caregivers") OR title("careg*") OR title("carer") OR title("care giv*") OR abstract("careg*") OR abstract("carer") OR abstract("care giv*")) AND ("aged" OR "older adult*" OR "older person*" OR "elderly") AND (MAINSUBJECT.EXACT("Systematic review") OR abstract("systematic review") OR title("systematic review")) AND la.exact("ENG")
Scopus	1/31/23	TITLE-ABS (("systematic review") AND ("careg*" OR "carer" OR "care giv*")) AND ("aged" OR "older adult*" OR "older person*" OR "elderly") AND (LIMIT-TO (PUBYEAR,2023) OR LIMIT-TO (PUBYEAR,2022) OR LIMIT-TO (PUBYEAR,2021) OR LIMIT-TO (PUBYEAR,2020) OR LIMIT-TO (PUBYEAR,2019) OR LIMIT-TO (PUBYEAR,2018)) AND (LIMIT-TO (LANGUAGE,"English"))
PubMed	1/30/23	(MAINSUBJECT.EXACT("Caregivers") OR tiab("careg*") OR tiab("carer") OR tiab("care giv*")) AND (MAINSUBJECT.EXACT("Systematic Review") OR tiab("systematic review")) AND ("aged" OR "older adult*" OR "older person*" OR "elderly")
PsycInfo	1/31/23	(MAINSUBJECT.EXACT("Caregivers") OR tiab("careg*") OR tiab("carer") OR tiab("care giv*")) AND (MAINSUBJECT.EXACT("Systematic Review") OR tiab("systematic review")) AND ("aged" OR "older adult*" OR "older person*" OR "elderly")
Sociological Abstracts	1/30/23	(abstract("systematic review") OR title("systematic review")) AND (mainsubject("Caregivers") OR abstract(carer) OR title(carer) OR abstract(careg*) OR title(careg*) OR abstract("care giv*") OR title("care giv*")) AND (mainsubject(elderly) OR aged OR elderly OR (older person) OR (older adult))<br>Date limit 1-30-18 to 1-30-2023<br>English
PAIS Index	1/30/23	(abstract("systematic review") OR title("systematic review")) AND (mainsubject("Caregivers") OR abstract(carer) OR title(carer) OR abstract(careg*) OR title(careg*) OR abstract("care giv*") OR title("care giv*")) AND (mainsubject(elderly) OR aged OR elderly OR (older person) OR (older adult))<br>Date limit 1-30-18 to 1-30-2023<br>English
EconLit	1/30/23	(abstract("systematic review") OR title("systematic review")) AND (mainsubject("Caregivers") OR abstract(carer) OR title(carer) OR abstract(careg*) OR title(careg*) OR abstract("care giv*") OR title("care giv*")) AND (mainsubject(elderly) OR aged OR elderly OR (older person) OR (older adult))<br>Date limit 1-30-18 to 1-30-2023<br>English

Appendix B

Authors & Year of Publication	Number of Studies in the Review	Date Range of Authors' Search	Review Objective(s) & Target Population	Number of Caregiver (CG) Participants (Total or Sample Size Range)	Intervention Types	Diagnosis-Driven, Intervention-Driven, or Both	Measurement Tools	Key CG Findings
Agustina et al., 2022	8	Inclusive of all publications through March 2022	To assess the impact of early palliative care on QoL of CGs of adults with cancer.	39-275	Multicomponent; Miscellaneous	Both	**Depression:** NR<br><br>**Burden:** NR<br><br>**QoL:** CQoL-C; QOLLTI-F	**Depression**: NR<br><br>**Burden**: 3/8 studies reported the early palliative care intervention studies showed a statistically significant reduction in burden.<br><br>**QoL**: 2/8 studies reported early palliative care intervention studies showed a statistically significant improvement in a QoL.
Akarusu et al., 2018	13	1990-2015	To assess the effectiveness of interventions to reduce depressive symptoms in ethnic minority CGs of adults with dementia.	1076	Psychoeducation; Counseling & Psychotherapy; Multicomponent	Diagnosis	**Depression**: CES-D; PHQ-9<br><br>**Burden:** N/A<br><br>**QoL:** N/A	**Depression**: Meta-analyses showed a statistically significant reduction in depressive symptoms with a small effect for psychosocial intervention vs. control group (SMD = −0.17, 95% CI = [−0.29, −0.05], $p = 0.005$, $I^2 = 0.0\%$,).<br><br>**Burden**: N/A<br><br>**QoL**: N/A
Andrades-Gonzalez et al., 2021	13	2009-2019	To assess the impact of e-health on the QoL of CGs of adults with stroke.	2003	Psychoeducation; Counseling; & Psychotherapy; Support groups	Both	**Depression:** CES-D; HADS; PHQ-9.<br><br>**Burden**: QASCI; OCBS; BCOS.	**Depression:** Meta-analyses showed a statistically significant reduction in depressive symptoms with a large effect for eHealth intervention vs. control group (SMD = 1.21, 95% CI = [-7.97, 3.87] $p = 0.00$, $I^2 = 0$).<br><br>**Burden:** Meta-analyses showed a statistically significant reduction in caring strain with a small effect for eHealth intervention vs. control group

							**QoL**: SSQOL-Pr; SF-36.	(SMD = 0.41, 95% CI = [-1.99, 2.8], $p = 0.015$, $I^2 = 0$).<br><br>**QoL**: Meta-analyses showed a statistically significant improvement in QoL with a large effect for eHealth intervention vs. control group (SMD = 0.14, 95% CI = [-0.05, 0.33], $p = 0.008$, $I^2 = 0\%$).
Baik et al., 2021	16	2010-2020	To assess trends in the use of physical activity interventions for CGs of adults with chronic diseases.	NR	Mindfulness-Based Interventions; Miscellaneous	Intervention	**Depression:** BDI<br><br>**Burden:** ZBS<br><br>**QoL**: N/A	**Depression:** 2/6 studies reported that the intervention showed a statistically significant improving in depressive symptoms, 2/6 studies reported no significant changes, and the remainder demonstrated inconsistent findings.<br><br>**Burden:** 5/5 studies reported the intervention showed a mixed results in burden (2 studies reported no significant effect on caregiving burden between groups but had positive impact within the intervention.<br><br>**QoL**: 1/5 studies reported significant effects on QoL within the intervention group**.**
Bennett et al., 2019	15	Inclusive of all publications through February 2018	To assess the impact of occupational therapy on BPSD and QoL for adults with dementia and on burden, depression and QoL for CGs of adults with dementia.	2063	Training of the CR with CG Involvement	Both	**Depression**: NR<br><br>**Burden**: ZBI<br><br>**QoL**: NR	**Depression**: Meta-analyses showed a non-significant reduction in depression with a small effect for occupational therapy vs. control group at immediate follow-up (SMD = −0.33, 95% CI = [−0.7, 0.04], $I^2 = 76\%$).<br><br>**Burden**: Meta-analyses showed a non-significant reduction in burden with a small effect for intervention group vs. control group at immediate follow up (SMD = −0.06, 95% CI = [−0.31, 0.18], $I^2 = 35\%$).<br><br>**QoL**: Meta-analyses showed a statistically significant improvement in QoL with a large effect in intervention group vs. control group at immediate follow up (SMD = 0.99, 95% CI = [0.66, 1.33], $p < 0.00010$, $I^2 = 2\%$).

Chacko et al., 2022	7	Inclusive of all publications through August 2020	To assess the efficacy of mindfulness-based cognitive therapy in reducing stress for CGs of adults with dementia.	12-113	Psychoeducation; Counseling & Psychotherapy; Mindfulness-Based Interventions Multicomponent	Both	**Depression**: CES-D; GDS-D; HADS; DASS<br><br>**Burden:** ZBI; CBI<br><br>**QoL**: SF-19; SF-12	**Depression**: 2/7 studies reported the mindfulness-based intervention showed a statistically significant improving in depressive symptoms.<br><br>**Burden**: 1/7 studies reported the mindfulness-based intervention showed a statistically significant improving in burden.<br><br>**QoL**: None of studies reported the interventions showed statistically significant reduction in perceived burden or distress.
Cheng et al., 2020	140	2006-2018	To examine the effects of nonpharmacological interventions for CGs of adults with dementia, with a focus on the comparative effects of psychological interventions relative to other types of intervention.	14- 518	Psychoeducation; Counseling & Psychotherapy; Multicomponent; Mindfulness-Based Interventions; Support Groups; Miscellaneous	Diagnosis	**Depression:** CESD; BDI; HADS<br><br>**Burden:** ZBI; NPI-D<br><br>**QoL:** N/A	**Depression:** Meta-analyses showed a statistically significant reduction in depressive symptoms with a small effect for experimental group vs. control group. ($g = -0.27$, 95% CI = [-0.34, -0.20], $p < 0.001$, $I^2 = 65\%$).<br><br>**Burden:** Meta-analyses showed a statistically significant reduction in burden with a small effect for experimental group vs. control group ($g = -.24$, 95% CI = [-0.31, -0.17], $p < 0.001$, $I^2 = 63\%$).<br><br>**QoL:** N/A
Cheng et al., 2022	142	1987 - 2020	To provide a synthesis of the overall intervention effects of CGs of adults with mild-moderate dementia	11-8095	Psychoeducation; Counseling & Psychotherapy; Multicomponent; Mindfulness-Based Interventions; Support Groups; Care Coordination & Case Management	Diagnosis	**Depression:** NPI; RMBPC<br><br>**Burden:** NR<br><br>**QoL**: QOL-AD; DQLI	**Depression**: Meta-analyses showed a statistically significant reduction in depressive symptoms with a small effect for experimental group vs. control group at immediate follow up ($g = -0.12$, 95% CI = [−0.24, −0.004], $p < 0.05$, $I^2 = 38\%$).<br><br>**Burden**: NR<br><br>**QoL**: Meta-analyses showed a statistically significant in improvement in QoL with a small effect for experimental group vs. control group [$g = 0.10$, 95% CI = [0.04, 0.16], $p < 0.001$, $I^2 = 9\%$).
Chin et al., 2022	11	2000-2021	To evaluate the	1276	Psychoeducation	Both	**Depression:** PHQ-9; GDS;	**Depression**: Meta-analyses showed a statistically significant reduction in depressive symptoms with a

			effectiveness of technology-based interventions in reducing psychological morbidities such as depressive symptomology, anxiety, burden and improving quality of life and self-efficacy in informal CGs of adults with stroke.				HADS; BDI<br><br>**Burden:** OCBS; CSI<br><br>**QoL**: BCOS; CarerQoL	small effect for technology-based interventions group vs. control group (d = −0.27, 95% CI = [−0.49, −0.05] p = 0.02, I^2 = 40%).<br><br>**Burden:** Meta-analyses showed a nonsignificant reduction in burden with a small effect for technology-based intervention group vs. control group (d = 0.05, 95% CI = [−0.26, 0.37], p = 0.74, I^2 = 13%).<br><br>**QoL**: Meta-analyses showed a nonsignificant improvement in QoL with a small effect for technology-based intervention group vs. control group (d = 0.12, 95% CI = [−0.14, 0.39], p = 0.36, I^2 = 13%).
Egan et al., 2018	8	Inclusive of all publications through 2016	To identify studies of Internet-based interventions designed to train and support CGs of adults with dementia.	900	Psychoeducation	Diagnosis	**Depression:** CES-D; BDI-II<br><br>**Burden:** ZBI<br><br>**QoL**: PQOL	**Depression**: 2/8 studies reported that internet-based interventions showed statistically significant reduction in depression between the intervention and control group.<br><br>**Burden**: No studies reported that internet-based interventions showed improvement in reducing burden. (NR)<br><br>**QoL**: 3/8 studies reported that internet-based interventions showed statistically significant improvement in QoL between the intervention and control group.
Frambes et al., 2018	14	2009-2016	To analyze recent caregiving literature for evidence of relationships between supportive interventions for CGs of adults with cancer.	NR	Psychoeducation; Counseling & Psychotherapy; Training of the CR with CG Involvement	Diagnosis	**Depression:** NR<br><br>**Burden:** NR<br><br>**QoL**: NR	**Depression**: 2/14 studies reported that supportive caregiver interventions showed statistically significant improvement in depression between the intervention and control group.<br><br>**Burden**: 1/14 studies reported that supportive caregiver intervention showed statistically significant reduction in burden between the intervention and control group.<br><br>**QoL**: 4/14 studies reported that interventions showed statistically significant improvement in QoL

								between the intervention and control group.
Frias et al., 2020	18	2005-2018	To assess the effectiveness of psychoeducational interventions with respect to burden, anxiety and depression in CG of adults with dementia living at home.	1697	Psychoeducation; Multicomponent	Both	**Depression:** CES-D<br><br>**Burden:** ZBI<br><br>**QoL**: N/A	**Depression**: 5/18 studies reported that the psychoeducational interventions showed statistically significant reduction in depressive symptoms between the intervention and control group.<br><br>**Burden**: 4/18 studies reported that the psychoeducational interventions showed statistically significant reduction in burden between the intervention and control group.<br><br>**QoL**: 1/18 studies reported that the psychoeducational interventions showed statistically significant improvement in QoL between the intervention and control group.
Han et al., 2022	15	Inclusive of all publications through May 2021	To assess the effects of MBIs on depressive symptoms, anxiety, stress, and quality of life in family CGs of adults living with dementia with subgroup analyses according to the type of control groups and the type of MBIs.	754	Mindfulness-Based Interventions; Counseling & Psychotherapy; Multicomponent	Both	**Depression:** POMS; CES-D, BDI; BDI-II; HRSD; WebNeuro; GDS<br><br>**Burden:** NR<br><br>**QoL:** SF-36; SF-12; WHOQOL-BREF; AqoL-8D	**Depression:** Meta-analyses showed a statistically significant reduction in depressive symptoms with moderate effect for MBIs vs. control group at the immediate posttest (SMD = 0.67, 95% CI = [0.48, 0.86], $p < 0.01$).<br><br>**Burden:** NR<br><br>**QoL:** Meta-analyses showed a statistically significant improvement in QoL with a large effect for MBIs vs. control group at the immediate posttest (SMD = 0.80, 95% CI = [0.27, 1.34], p = 0.003).
He et al., 2022	31	Inclusive of publications through July 2020	To examine whether multi-component interventions for informal CGs of adults with	3939	Multicomponent	Both	**Depression:** CES-D; SDS; GDS; PHQ; BDI-II; Symptom Checklist 90<br><br>**Burden:** NR	**Depression:**<br>Meta-analyses showed a statistically significant reduction in depression with small effect in multi-component interventions vs. control group (SMD = −0.29, 95% CI = [−0.46, −0.11], p = 0.001, I^2 = 0%).<br><br>**Burden:** Meta-analyses showed a statistically

			dementia are effective on positive and negative aspects of CG wellbeing.				**QoL (Referred to as Subjective Well-Being)**: SPACE; NHP; PCI; PANAS; CarerQoL; VAS	significant reduction in burden with small effect in multi-component interventions vs. control group post-intervention (SMD = −0.34, 95% CI = [−0.53, −0.16], p = 0.0003, I^2 = 60%).<br><br>**QoL:**<br>Meta-analyses showed a statistically significant improvement in subjective well-being with small effect in multi-component interventions vs. control group (SMD = 0.41, 95% CI = [0.28, 0.54], p < 0.001, I^2 = 25%).
Hovadick et al., 2021	6	2009-2020	To review the effectiveness and limitations of interventions in improving the well-being of family CGs of adults with end stage renal disease on hemodialysis or peritoneal dialysis.	38-105	Support Groups; Multicomponent	Diagnosis	**Depression:** NR<br><br>**Burden:** ZBI; ZBS; CBI<br><br>**QoL**: SF-36	**Depression:** NR<br><br>**Burden:** 5/5 studies demonstrated **s**tatistically significant improvements in burden levels were identified in the intervention group vs. the control group.<br><br>**QoL:** In 1 study, no significant differences were found in QoL between the intervention and control group.
Kishita et al., 2018	31	2006-2016	To update the literature on interventions for CGs of adults with dementia and evaluate the efficacy of psychoeducational programs and psychotherapeutic interventions on key mental health	NR	Psychoeducation; Counseling & Psychotherapy	Diagnosis	**Depression:** CESD; GDS; MADRS<br><br>**Burden:** ZBI; CBI<br><br>**QoL**: WHOQoL-BREF; SF-12; GWS	**Depression:**<br>Meta-analyses showed a nonsignificant reduction in depression with small effect for psychoeducation-skill building interventions vs. control group, and a statistically significant, moderate effect on depression for CBT-based psychotherapeutic interventions compared to control group:<br><br>Psychoeducation-skill building: (g = 0.12, p = 0.14, 95% CI = [−0.04 to 0.29])<br>CBT: (g = 0.53, p < 0.01; 95% CI [0.22 to 0.84]).<br><br>**Burden:**<br>Meta-analyses showed a nonsignificant reduction in burden with small effect for

			outcomes.					psychoeducation-skill building interventions vs. control group, and a nonsignificant reduction in burden with small effect for CBT-based psychotherapeutic interventions vs. control group:<br><br>Psychoeducation-skill building (g = 0.18, p < 0.01; 95% CI = [0.06 to 0.29]); CBT (g = 0.09, 95% CI = [−0.03 to 0.21, p = 0.14]).<br><br>**QoL:**<br>Meta-analyses showed a nonsignificant reduction in QoL with moderate effect for psychoeducation-skill building interventions vs. control group:<br>Psychoeducation-skill building (g = 0.60, p = 0.15, 95% CI = [−0.21 to 1.42]).
Knowles et al., 2021	20	Inclusive of all publications through April 2020	To inform health care teams on an evidence-based approach to supporting the CGs of adults with heart disease.	1776	Psychoeducation; Miscellaneous	Diagnosis	**Depression:** HADS; CES-D; BAI<br><br>**Burden:** ZBI; CBS; CBQ-HF<br><br>**QoL**: SF-12; SF-36	**Depression:**<br>Meta-analyses showed a nonsignificant reduction in depression with moderate effect for interventions vs. control group at 2 to 4 months post-intervention (g = −0.64, 95% CI = [−1.34, 0.06]).<br><br>**Burden:**<br>Meta-analyses showed a nonsignificant reduction in burden with moderate effect for interventions vs. control group at 2 to 4 months post-intervention (g = −0.51, 95% CI = [−2.71, 1.70]).<br><br>**QoL:**<br>Meta-analyses showed a nonsignificant improvement in physical quality of life with moderate effect for interventions vs. control group at 2 to 4 months post-intervention (g = 0.178, 95% CI = [−0.09, 0.45]).
Kustanti et al. 2021	19	Inclusive of all publications through January 2020	To summarize and synthesize the effectiveness of bereavement support for adult family CGs of adults receiving	2,690	Miscellaneous	Intervention	**Depression:** NR<br><br>**Burden:** N/A<br><br>**QoL**: N/A	**Depression:**<br>Meta-analyses showed a statistically significant reduction in depression with a small effect in bereavement support vs. control group (g = -0.252, 95% CI = [-0.406, -0.098], p = 0.001, I^2 = 63.773%).<br><br>**Burden:** N/A<br><br>**QoL:** N/A

			palliative care.					
Lee et al., 2020b	26	2005-2017	To evaluate the effectiveness of different types of psychosocial interventions on the health-related quality of life among CGs of adults with dementia and to present an overview and assessment of the quality of the most recent intervention studies.	3,906	Psychoeducation; Counseling & Psychotherapy; Multicomponent; Support Groups; Care Coordination & Case Management; Miscellaneous	Diagnosis	**Depression:** N/A<br><br>**Burden:** N/A<br><br>**QoL**: WHOQoL-BREF; QoLQ; Dementia Quality of Life; SF−12; SF−36; Quality of Life, Alzheimer Disease, Health-related Quality of Life−15D; Cantril Ladder, QOLS; EQ−5D-5L; VAS	**Depression:** N/A<br><br>**Burden:** N/A<br><br>**QoL:**<br>Meta-analyses showed a statistically significant improvement in QoL with a small effect in multicomponent interventions vs. control group immediately post-intervention (g= 0.255, 95% CI = [0.054, 0.457], p = 0.013).
Lee et al., 2020a	31	2007-2017	To examine the quality and effectiveness of interventions to reduce depressive symptoms reported by CGs of adults with dementia.	4039	Psychoeducation; Counseling & Psychotherapy; Multicomponent; Mindfulness-Based Interventions, Support Groups; Miscellaneous	Diagnosis	**Depression:** CES-D; HADS; GDS; BDI; MADRS; CSDD<br><br>**Burden:** N/A<br><br>**QoL**: N/A	**Depression:**<br>Meta-analyses showed a reduction in depressive symptoms with small to large effect (see below) for interventions vs. control group:<br><br>CBT: (SMD = -0.905, 95% CI = [-1.622, -0.187], p = 0.013, I^2 = 94.78%);<br>Psychoeducational: (SMD =-0.244, 95% CI = [-0.395, 0.092], p = 0.002, I^2 = 0%);<br>Emotional Support Intervention: (SMD = -0.123, 95% CI = [-0.344, 0.097], p = 0.272, I^2 = 0%);<br>Cognitive Rehab: (SMD = -0.104, 95% CI = [-0.240, 0.031], p = 0.130, I^2 = 0%);<br>Mindfulness Intervention: (SMD = -0.578, 95% CI = [-0.881, -0.275], p < 0.001, I^2 = 0%);<br>Multicomponent Intervention: (SMD = -0.123, 95% CI = [-0.249, 0.003], p = 0.055, I^2 = 0%).<br><br>**Burden:** N/A

								**QoL:** N/A
Liu et al., 2022	8	Inclusive of all publications through August 2020	To systematically evaluate the effectiveness of psychological intervention durations and different psychological interventions on treating depression in primary CGs of adults with Alzheimer's disease.	857	Psychoeducation; Counseling & Psychotherapy; Multicomponent	Diagnosis	**Depression:** SDS; CES-D<br><br>**Burden:** N/A<br><br>**QoL**: N/A	**Depression:** Meta-analyses showed a significant pooled absolute reduction in depression for interventions vs. control group at 6 months (MD = −6.65, 95% CI = [−12.91, −0.39], $p = 0.04$, $I^2 = 96\%$).<br><br>**Burden:** N/A<br><br>**QoL:** N/A
Lee et al., 2021	15	NR	To synthesize the effectiveness of psychosocial interventions on CGs of adults with advanced cancer, in comparison with usual care, on CGs quality of life (QoL), anxiety, and depression symptoms.	**Depression:** 345<br><br>**QoL:** 729	Psychoeducation; Multicomponent; Support Groups; Miscellaneous	Both	**Depression:** HADS; PROMIS; CESD; DASS; BDI-II; SCL-92; PHQ9<br><br>**Burden:** N/A<br><br>**QoL**: CQOL-C; COH QOL; CQLI-R; QOLLTI-F	**Depression:** Meta-analyses showed a statistically significant reduction in depression with moderate effect in psychosocial interventions vs. control group immediately post-intervention (SMD = − 0.65, 95% CI = [-0.98, -0.31], $p < 0.001$, $I^2 = 40\%$).<br><br>**Burden:** N/A<br><br>**QoL:** Meta-analyses showed a statistically significant improvement in QoL with moderate effect in psychosocial interventions vs. control group immediately post-intervention (SMD = 0.45, 95% CI = [0.09, 0.81], $p = 0.01$, $I^2 = 78\%$).
Lucero et al., 2019	12	Inclusive of all publications through July 2017	To evaluate the state of the science of ICT interventions on the health	32-250	Psychoeducation; Counseling & Psychotherapy; Multicomponent, Support Groups; Care	Both	**Depression:** NR<br><br>**Burden:** NR<br><br>**QoL**: N/A	4/8 information and communication technology interventions reported statistically significant effects on CG outcomes, including reductions in **depression** and **burden**.

			of informal CGs of adults with dementia.		Coordination & Case Management; Multicomponent; Miscellaneous			
Martensson et al., 2023	15	Inclusive of all publications through November 2021	To examine the effectiveness of psychological interventions for symptoms of depression and mental health related secondary outcomes among informal CGs of community-dwelling older adults with dementia, MCI, stroke, and mixed diagnoses, alongside potential clinical and methodological moderators.	1270	Psychoeducation; Counseling & Psychotherapy; Multicomponent	Intervention	**Depression:** CES-D; GDS; HADS; PHQ-9; BDI-II<br><br>**Burden:** N/A<br><br>**QoL**: N/A	**Depression:**<br>Meta-analyses showed a significant reduction in depression symptoms with small effect for psychological interventions vs. control group (g = −0.49, 95% CI = [−0.79, −0.19], p = 0.001, I^2 = 83.42%).<br><br>**Burden:**<br>Meta-analyses showed a significant reduction in burden with small effect for psychological interventions vs. control group (g = −0.35, 95% CI = [−0.55, −0.15], (p = 0.001).<br><br>**QoL:** NR
Martis et al., 2023	13	2010-2021	To synthesize evidence on yoga therapy and determine its effects on CG stress and mental health among CGs of adults with dementia.	522	Mindfulness-Based Interventions	Both	**Depression:** NR<br><br>**Burden:** NR<br><br>**QoL**: NR	**Depression:**<br>Meta-analyses showed a statistically significant reduction in depressive symptoms with small effect for interventions vs. control group (SMD = −0.33%, 95% CI = [− 0.27, − 0.65], p = 0.54, I^2 = 0%).<br><br>**Burden:**<br>Meta-analyses showed a statistically significant reduction in burden with large effect for interventions vs. control group (SMD = −1.07%, 95% CI = [0.05, − 0.23], p < 0.00001, I^2 = 98%).

								**QoL:** Insufficient data for meta-analysis.
Moore et al., 2020	11	Inclusive of all publications through April 2018	To explore whether interventions incorporating education regarding the progressive nature of dementia increased CGs of adults with dementia understanding of dementia and improved mental health and burden.	16-292	Psychoeducation; Multicomponent	Both	**Depression:** BDI; CES-D; MADRS; BSI<br><br>**Burden:** FCBI; CBI<br><br>**QoL**: ZBI	**Depression:**<br>Meta-analyses showed a statistically significant reduction in depressive symptoms with small effect for pooled education interventions at follow-up (SMD = -0.48, 95% CI = [-0.82, 0.14], p = 0.006, I^2 = 0%).<br><br>**Burden:**<br>Meta-analyses showed a nonsignificant reduction in burden with small effect for education interventions at follow-up (SMD = -0.31, 95% CI = [-0.64, 0.03], p = 0.07, I^2 =43%).<br><br>**QoL:**<br>N/A
Mou et al., 2021	11	Inclusive of all publications through April 2020	To evaluate the current evidence on supporting the dyadic psychoeducational intervention for the functional and psychosocial health of stroke survivors' and their family CGs.	1578	Psychoeducation; Multicomponent	Both	**Depression:** GDS; HADS<br><br>**Burden:** CBI; ZBI<br><br>**QoL**: AQoL-6D; EQ-5D-3L	**Depression:**<br>Meta-analysis showed a nonsignificant reduction in depression with moderate effect for dyadic intervention immediately post-test (SMD = −0.50, 95% CI = [−1.07, 0.08], p = 0.09).<br><br>**Burden:**<br>Meta-analysis showed a significant reduction in burden with small effect immediately post-test (SMD = −0.25, 95% CI = [−0.50, −0.01], p = 0.04).<br><br>**QoL:**<br>Insufficient data for meta-analysis.
Sun et al., 2022b	85	Inclusive of all publications through March 2021	To compare and rank the efficacy of different non-pharmacological interventions on	7672 (total)<br><br>**Depression:** 5,882<br><br>**QoL:** NR<br><br>**Burden:**	Psychoeducation; Counseling & Psychotherapy, Multicomponent; Mindfulness-Based Interventions; Support Groups,	Both	**Depression:** CES-D; BDI; HDRS; GDS; MADRS; MAACL (depression subscale)	**Depression:**<br>Meta-analyses showed a statistically significant reduction in depression symptoms with a moderate to large effect (see below) for six intervention types vs control group:<br>Acceptance and Commitment Therapy (SMD = −1.60, 95% CI = [−2.81, −0.39]);<br>Behavioral Activation (SMD = −1.37, 95% CI = [−1.90 to

			depression, anxiety, quality of life and burden for CGs of people with dementia.	4,929	Care Coordination & Case Management		**Burden:** ZBI; MBCB; FCBI; BSFC<br><br>**QoL:** SF-36; WHOQOL-BREF; EQLS	− 0.85]);<br>Mindfulness-based Intervention (SMD = −0.78, 95% CI = [−1.16, −0.41]);<br>Multicomponent Intervention (SMD = −0.56, 95% CI = [−0.92 to −0.21]);<br>Psychoeducation (SMD = −0.55, 95% CI = [−0.76, −0.34]);<br>Cognitive Behavioral Therapy (SMD = −0.53, 95% CI = [−0.92, −0.14]).<br><br>**Burden:**<br>Meta-analyses showed a statistically significant reduction in burden with a small to large effect (see below) for three interventions vs. control group:<br>Case management (SMD = −1.26, 95% CI = [−2.12, −0.40]); Psychoeducation (SMD = − 0.60, 95% CI = [− 0.87, − 0.33]);<br>Multicomponent Intervention (SMD = − 0.36, 95% CI = [− 0.71, − 0.01]).<br><br>**QoL:**<br>Meta-analyses showed a statistically significant improvement in QoL with moderate effect for the support group intervention vs. control group (SMD = 0.69, 95% CI = [0.16, 1.22]).
Sun et al., 2022a	37	Inclusive of all publications through	To examine the most effective delivery format for CBT for CGs of adults with dementia via a network meta-analysis.	4,191	Counseling & Psychotherapy	Both	**Depression:** BDI-I; BDI-II; CES-D; HADS; GDS; MADRS; QD-R, SDS<br><br>**Burden:** N/A<br><br>**QoL:** N/A	**Depression:**<br>Meta-analyses showed statistically significant reduction in depressive symptoms with a large effect for CBT interventions via three delivery formats vs. control group:<br>Internet (SMD = − 1.45, 95% CI = [− 2.31, − 0.28]);<br>Telephone (SMD = − 1.28, 95% CI = [− 1.78, − 0.53]);<br>Individual (SMD = − 1.19, 95% CI = [− 2.29, − 0.31]).<br><br>**Burden:** N/A<br><br>**QoL:** N/A
Teahan et al., 2020	22	2006-2016	To review and synthesize findings of the	**Depression:** 1,856<br><br>**Burden:** 1,485	Psychoeducation; Counseling & Psychotherapy; Multicomponent; Miscellaneous	Both	**Depression:** CES-D; GHQ (depression subscale); HADS	**Depression:**<br>Meta-analyses showed a statistically significant reduction in depression with a small effect pre- and post-psychosocial interventions (SMD = −0.36, 95% CI = [−0.60, −0.13], p = 0.002).

			effectiveness of psychosocial interventions aimed at improving outcomes for CGs of adults with dementia.	**QoL:** 201			**Burden:** ZBI; FCBI; CBI; MBCB<br><br>**QoL:** WHO-QoL; WHOQOL-BREF	**Burden:**<br>Meta-analyses showed a statistically significant reduction in burden with a small effect for psychosocial interventions vs. control group (SMD = -0.34, [95% CI = −0.59, −0.09], p = 0.007).<br><br>**QoL:**<br>Meta-analyses showed a nonsignificant improvement in QoL with a moderate effect size for psychosocial interventions vs. control group (SMD = 0.63, 95% CI = −0.16, 1.43, p=0.12).
Wang et al., 2020	9	Inclusive of all publications through July 2019	To explore what types of bibliotherapy have been used for improving the mental well-being of informal CGs of adults with neurocognitive disorders, and the effect on mental well-being out- comes.	1,036<br><br>**Depression:** NR<br><br>**Burden:** N/A<br><br>**QoL:** N/A	Miscellaneous; Multicomponent	Both	**Depression:** CES-D; BDI; BDI-II<br><br>**Burden:** N/A<br><br>**QoL:** N/A	**Depression:**<br>Meta-analyses showed a statistically significant reduction in depression with a moderate effect for video and web-based bibliotherapy vs. control group (SMD = -0.74, 95% CI = [-1.47, -0.01], p = 0.05, I^2 = 94%).<br><br>**Burden:** N/A<br><br>**QoL:** N/A
Wang et al., 2021	29	Inclusive of all publications through July 2021	To explore which psychosocial interventions exist for informal CGs of adults with early dementia. To address: (1) what components of psychosocial interventions are effective	**Depression:** NR<br><br>**Burden:** NR<br><br>**QoL:** NR	Psychoeducation; Counseling & Psychotherapy; Multicomponent; Mindfulness-Based Interventions; Support Groups	Both	**Depression:** NR<br><br>**Burden:** NR<br><br>**QoL:** NR	3 training interventions and 10/12 multi-component interventions that shared the common intervention component of training, reported statistically significant effects, including fewer **depressive symptoms**, improved **QoL**, and reduced CG **burden.**<br><br>14/16 multicomponent interventions that shared the common intervention component of education reported benefits including improved **QoL**, reduced **depressive symptoms** and **burden**.<br><br>6 studies of multicomponent interventions that shared the common intervention components of education + social support showed beneficial effects on **depressive symptoms**, perceived **burden** and **QoL.**

			for informal CGs of adults with early dementia, (2) what theories underpin these interventions, and (3) What CG's needs were assessed in these interventions.					9/10 multicomponent interventions that shared the common interventions of education + training reported improved **QoL** and reduced **burden.**
Wiegelmann et al., 2021	48	2009-2018	To provide an update on high quality psychosocial intervention studies on mental health promotion for informal CGs of adults with dementia, describing intervention effects on key mental health outcomes.	**Depression:** NR<br><br>**Burden:** NR<br><br>**QoL:** NR	Psychoeducation; Counseling & Psychotherapy; Multicomponent; Miscellaneous	Both	**Depression:** CES-D most common (62.5%); other tools NR<br><br>**Burden:** ZBI most common (69.2% of studies); other tools NR<br><br>**QoL:** SF-12 and SF-36 most common; other tools NR	**Depression:** 9/24 studies assessing depression reported statistically significant improvement in CG depression. Intervention types included psychoeducation (4 studies showed improvement), counseling (1 w/ improvement) and CBT (4 w/ improvement).<br><br>**Burden:** 12/26 studies assessing burden reported significant improvement in subjective burden. Intervention types included psychoeducation (5 w/ improvement), leisure and physical activity (3 w/improvement), counseling (2 w/improvement) and CBT (2 w/improvement).<br><br>**QoL:** 5/23 studies assessing quality of life reported statistically significant improvement. Intervention types included psychoeducation (3 w/ improvement), leisure and physical activity (1 w/ improvement), and counseling (1 w/improvement).
Williams et al., 2019	34	1999-2018	To examine the published evidence for interventions designed to reduce levels of burden for CGs of adults with dementia.	17-406	Psychoeducation, Counseling & Psychotherapy; Multicomponent; Miscellaneous	Diagnosis	**Depression:** N/A<br><br>**Burden:** ZBI; ZBS; FCBI; RMBPC; CBI; CSI<br><br>**QoL:** N/A	**Depression:** N/A<br><br>**Burden:**<br>Meta-analyses showed a statistically significant reduction in burden with a small effect for interventions vs. control group (SMD = -0.18, 95% CI = [-0.30, -0.05], p = 0.005, I^2 = 63%).<br><br>Sub-analysis showed a statistically significant reduction in burden with a small effect for multi-

								component interventions vs. control group (SMD = -0.22, 95% CI = [-0.41, -0.03], p = 0.02, I^2 = 63%).<br><br>**QoL:** N/A
Zabihi et al., 2020	12	Inclusive of all publications through 2018	To establish the treatment effect of behavioral activation for depression for CGs of adults with dementia by reviewing worldwide evidence, report on its quality and assess whether study parameters modified the treatment effect.	**Depression:** 1,904<br><br>**Burden:** N/A<br><br>**QoL:** N/A	Counseling & Psychotherapy; Multicomponent	Both	**Depression:** CES-D; MAACL; SADS; DASS-21; BDI-II; SCID-CV; ATQ-N<br><br>**Burden:** N/A<br><br>**QoL:** N/A	**Depression:**<br>Meta-analyses showed a statistically significant reduction in depressive symptoms with a moderate to large effect (see below) pre- and post-behavioral activation interventions at 4-14 weeks (SMD -0.68, 95% CI = [-1.14, -0.22], I^2 = 89%) and at 1-year (SMD -0.99, 95% CI = [-1.26, -0.71], I^2 = 92%).<br><br>**Burden:** N/A<br><br>**QoL:** N/A
Zhao et al., 2019	6	Inclusive of all publications through 2018	To examine the effect of internet-based interventions on the mental health outcomes of CG of adults with dementia and to explore which components of the Web-based interventions play an important role.	**Depression:** 626<br><br>**Burden:** 184<br><br>**QoL:** NR	Psychoeducation; Multicomponent	Both	**Depression:** CES-D; BDI-II<br><br>**Burden:** ZBS<br><br>**QoL:** EuroQoL; WHO-QoL	**Depression:**<br>Meta-analyses showed a statistically significant reduction in depression scores with a small effect pre- and post- web-based interventions (SMD = -0.23, 95% CI = [−0.38 to −0.07], p = 0.005, I^2 = 0%).<br><br>**Burden:** Full meta-analyses findings not reported.<br><br>**QoL:** Full meta-analyses findings not reported.
Zheng et al., 2021	12	2004-2019	To explore the effects of	10-133 (750 total	Psychoeducation; Counseling &	Both	**Depression:** HADS; CES-	**Depression:** 4/10 studies reported that spirituality-integrated interventions showed statistically

			spirituality-integrated interventions for informal CGs of terminally ill patients.	CGs)	Psychotherapy, Multicomponent; Mindfulness-Based Interventions; Support Groups; Miscellaneous		D; POMS-B; BDI-II; BHS; PROMIS<br><br>**Burden:** ZBI; CBS<br><br>**QoL:** WHOQOL-BREF; CQOLC; SF-36; LASA; SWLS; PEACE	significant reduction in depression and anxiety.<br><br>**Burden:** 2/9 studies reported the interventions showed statistically significant reduction in perceived burden or distress.<br><br>**QoL:** 3/5 studies reported the interventions showed improvement in overall QoL or some domains of QoL.
Zhou et al., 2022	17	Inclusive of all publications through 2021	To systematically review the effect of CBT in terms of improving the QoL and negative emotion of CGs of adults with cancer.	26-476 (2,348 total CGs)<br><br>Individual outcome sample sizes NR	Counseling & Psychotherapy	Both	**Depression:** POMS; CES-D; HADS; HAMD; DASS-21<br><br>**Burden:** N/A<br><br>**QoL:** CQOL-C; SF-36; WHOQOL; COH-QOL	**Depression:** Meta-analyses showed a statistically significant reduction in depression scores with a small effect for the CBT intervention vs. control group (SMD = −0.32, 95% CI = [−0.56, −0.07], $p = 0.010$, $I^2 = 67.0\%$).<br><br>**Burden:** N/A<br><br>**QoL:** Meta-analyses showed a nonsignificant difference in QoL with a small effect for CBT intervention vs. control group (SMD = 0.28, 95% CI = [-0.09, 0.65], $p < 0.001$, $I^2 = 90.1\%$).
Zhu et al., 2021	11	Inclusive of all publications through December 31, 2019	To evaluate the major mental health outcomes for CGs of adults with dementia when using psychoeducational programs and psychotherapeutic interventions.	**Depression:** NR<br><br>**Burden:** NR<br><br>**QoL:** NR	Psychoeducation; Counseling & Psychotherapy; Support Groups;<br><br>*(limited information reported beyond method of delivery)*	Both	**Depression:** CES-D<br><br>**Burden:** ZBI<br><br>**QoL:** ICECAP-O; Euro-QoL	**Depression:** Meta-analyses showed a statistically significant reduction in depression with a small effect for interventions vs. control group (SMD = -0.34, 95% CI = [-0.54, -0.14], $p < 0.01$, $I^2 = 55\%$).<br><br>**Burden:** Meta-analyses showed a nonsignificant reduction in burden with a negligible effect for intervention vs. control group (SMD = -0.04, 95% CI = [-0.23, 0.14], $p = 0.65$, $I^2 = 0\%$).<br><br>**QoL:** Meta-analyses showed a nonsignificant improvement in QoL with a small effect for intervention vs. control group (SMD = 0.23, 95% CI = [-0.04, 0.5], $p = 0.09$, $I^2 = 19\%$).
Zulkifley et al., 2020	8	1998-2018	To provide a narrative synthesis on	662	Psychoeducation; Multicomponent	Both	**Depression:** CES-D; MADRS; BSI	**Depression:** 3/6 studies found significant reduction in depressive symptoms on the intervention group.

			the methods of educational intervention implementation and the outcomes of each intervention for CGs of adults with dementia.				(6-item depression subscale)<br><br>**Burden:** ZBI; MCSI<br><br>**QoL:** N/A	**Burden:** 2/5 studies showed significant reduction in burden after educational intervention.<br><br>**QoL:** N/A

* For meta-analyses, the total analyzed sample is reported, for narrative analyses, the sample ranges are reported.

CBT	Cognitive Behavioral Therapy
CG	Caregiver
d	Cohens D
g	Hedges G
MD	Mean Difference
SMD	Standardized Mean Difference
QoL	Quality of Life
95% CI	95% Confidence Interval

**Measurement Tool**	**Abbreviation**
Depression	
Center for Epidemiologic Studies Depression Scale	CES-D, CESD
The Patient Health Questionnaire (9-question)	PHQ, PHQ-9
Profile of Mood States, Profile of Mood States-Brief	POMS, POMS-B
Beck Depression Inventory/Beck Depression Inventory II	BDI, BDI-II
Beck Hopelessness Scale	BHS
Hamilton Rating Scale for Depression/Hamilton Depression Rating Scale/Hamilton Depression Scale	HRSD/HDRS/HAMD
WebNeuro	n/a
Geriatric Depression Scale	GDS, GDS-D
Montgomery and Asberg Depression Rating Scale	MADRS
Multiple Affect Adjective Checklist Hostility	MAACL
Hospital Anxiety and Depression Scale	HADS
Depression Questionnaire – Reduced Form	QD-R
Self-Rating Depression Scale	SDS
Depression Anxiety Stress Scales	DASS, DASS-21
General Health Questionnaire	GHQ

Patient-Reported Outcomes Measurement Information System	PROMIS
Revised Memory and Behavior Problems Checklist	RMBPC
Schedule for Affective Disorders and Schizophrenia	SADS
Structured Clinical Interview for DSMIV	SCID-CV
Automatic Thoughts Questionnaire-Negative	ATQ-N
Symptom Checklist – 92 Instrument	SCL-92
PROMIS scale for anxiety/depression	PROMIS
Brief Symptom Inventory	BSI
Beck Anxiety Inventory	BAI
Cornell Scale for Depression in Dementia	CSDD
Caregiver Burden	
Zarit Burden Interview/Scale	ZBI/ZBS
Montgomery Borgatta Caregiver Burden Scale	MBCB
Family Caregiving Burden Inventory/Caregiver Burden Inventory	FCBI/CBI
Burden Scale for Family Caregivers	BSFC
Oberst Caregiver Burden Scale	OCBS
Caregiver Burden Scale (Elmstahl)	CBS
Caregiver Burden Questionnaire—Heart Failure	CBQ-HF
Revised Memory and Behavior Problems Checklist	RMBPC
Caregiver Strain Instrument	CSI
Modified Caregiver Strain Index	MCSI
Questionário de Avaliação de Sobrecarga do Cuidador Informal	QASCI
Bakas Caregiving Outcomes Scale	BCOS
Neuropsychiatric Inventory Caregiver Distress Scale	NPI-D
Copenhagen Burnout Inventory	CBI
Quality of Life (QoL)	
Caregiver Quality of Life – Cancer Assessment	CQoL-C/CQOLC
Quality of Life in Life-Threatening Illness – Family Caregiver Questionnaire	QOLLTI-F
Short Form Health Survey (12-question, 36-question)	SF-12, SF-36
World Health Organization Quality of Life	WHO-QoL
World Health Organization Quality of Life – Brief Version	WHOQOL-BREF
Assessment of Quality of Life – 8 Dimension, 6 Dimension	AQoL-8D, AQoL-6D
European Quality of Life Survey- 5 Dimension	EQLS, Euro QoL, EQ-5D-3L
City of Hope Quality of Life Scale	COH QOL
Caregiver Quality of Life Index- Revised	CQLI-R
Quality of Life in Alzheimer's Disease Scale	QOL-AD
Dementia Quality of Life Instrument	DEMQOL

Linear Analog Self-Assessment	LASA
Satisfaction with Life Scale	SWLS
Peace, Equanimity, and Acceptance in the Cancer Experience Questionnaire	PEACE
Investigation Choice Experiments for the Preferences of Older People	ICECAP-O
Stroke Specific Quality of Life Scale	SSQOL-PR
Dermatology Life Quality Index	DLQI
Care-related Quality of Life Instrument	CarerQoL
Perceived Quality of Life Scale	PQOL
Scale for Positive Aspects of Caregiving Experience	SPACE
Nottingham Health Profile	NHP
Perceived Change Index	PCI
Positive Affect and Negative Affect Schedule	PANAS
Visual Analogue Scale	VAS
Quality of Life Questionnaire	QoLQ
Quality of Life Scale	QOLS

**Table of Evidence Legend**	
**Diagnosis-Driven**	Inclusion criteria of review includes only limited, specified diagnoses.
**Intervention-Driven**	Review's inclusion criteria of includes specified intervention types.

Appendix C: AMSTAR 2 Ratings

	**AMSTAR 2 Ratings**						
**AMSTAR 2 Questions**	Augustina et al., 2022	Akarusu et al., 2018	Andrades-Gonzalez et al., 2021	Baik et al., 2021	Bennett et al., 2019	Chacko et al., 2022	Cheng et al., 2020
Q1: Did the research questions and inclusion criteria for the review include the components of PICO?	Yes	Yes	Yes	Yes	Yes	Yes	Yes
*Q2: Did the report of the review contain an explicit statement that the review methods were established prior to the conduct of the review and did the report justify any significant deviations?	Yes	Yes	Yes	Partial yes	Yes	Yes	Yes
Q3: Did the review authors explain their selection of the study designs for inclusion in the review?	No	No	No	Yes	No	No	No

*Q4: Did the review authors use a comprehensive literature search strategy?	Partial yes	Partial yes	Partial yes	Partial yes	Partial yes	Partial yes	Yes
Q5: Did the review authors perform study selection in duplicate?	Yes	Yes	Yes	Yes	Yes	Yes	Yes
Q6: Did the review authors perform data extraction in duplicate?	Yes	Yes	Yes	Yes	Yes	Yes	Yes
Q7: Did the review authors provide a list of excluded studies and justify the exclusions?	No	No	No	No	No	No	No
Q8: Did the review authors describe the included studies in adequate detail?	Partial yes	Yes	Partial yes	Yes	Partial yes	Partial yes	Partial yes
*Q9: Did the review authors use a satisfactory technique for assessing the risk of bias (RoB) in individual studies that were included in the review?	No	Partial yes	Yes	Yes	Yes	Yes	Yes
Q10: Did the review authors report on the sources of funding for the studies included in the review?	No	No	No	No	No	No	No
*Q11: If meta-analysis was performed did the review authors use appropriate methods for statistical combination of results?	N/a	Yes	Yes	Yes	Yes	N/a	Yes
Q12: If meta-analysis was performed, did the review authors assess the potential impact of RoB in individual studies on the results of the meta-analysis or other evidence synthesis?	N/a	No	No	Yes	Yes	N/a	Yes
*Q13: Did the review authors account for RoB in individual studies when interpreting/discussing the results of the review?	No	Yes	No	No	Yes	Yes	Yes
Q14: Did the review authors provide a satisfactory explanation for, and discussion of, any heterogeneity observed in the results of the review?	No	No	Yes	No	Yes	No	Yes
*Q15: If they performed quantitative synthesis did the review authors carry out an adequate investigation of publication bias and discuss its likely impact on the results of the review?	N/a	Yes	Yes	Yes	Yes	N/a	Yes
Q16: Did the review authors report any potential sources of conflict of interest, including any funding they received for conducting the review?	Yes	Yes	No	Yes	Yes	Yes	No
**Overall Rating**	Critically low	Moderate	Low	Low	Moderate	Moderate	Critically low
	**AMSTAR 2 Ratings**						

**AMSTAR 2 Questions**	Cheng et al., 2022	Chin et al., 2022	Egan et al., 2018	Frambes et al., 2018	Frias et al., 2020	Han et al., 2022	He et al., 2022
Q1: Did the research questions and inclusion criteria for the review include the components of PICO?	Yes	Yes	Yes	Yes	Yes	Yes	Yes
*Q2: Did the report of the review contain an explicit statement that the review methods were established prior to the conduct of the review and did the report justify any significant deviations?	Yes	Yes	Partial yes	Partial yes	Yes	No	Yes
Q3: Did the review authors explain their selection of the study designs for inclusion in the review?	No	No	No	No	No	No	No
*Q4: Did the review authors use a comprehensive literature search strategy?	Yes	Yes	Yes	Partial yes	Partial yes	Partial yes	Partial yes
Q5: Did the review authors perform study selection in duplicate?	Yes	Yes	No	No	Yes	No	Yes
Q6: Did the review authors perform data extraction in duplicate?	Yes	Yes	No	No	Yes	No	No
Q7: Did the review authors provide a list of excluded studies and justify the exclusions?	No	No	No	No	No	No	No
Q8: Did the review authors describe the included studies in adequate detail?	Partial yes	Yes	Partial yes	Partial yes	Partial yes	Partial yes	Partial yes
*Q9: Did the review authors use a satisfactory technique for assessing the risk of bias (RoB) in individual studies that were included in the review?	Yes	Yes	Yes	Yes	Yes	Yes	Yes
Q10: Did the review authors report on the sources of funding for the studies included in the review?	No	No	No	No	No	No	No
*Q11: If meta-analysis was performed did the review authors use appropriate methods for statistical combination of results?	Yes	Yes	Yes	N/a	N/a	Yes	Yes
Q12: If meta-analysis was performed, did the review authors assess the potential impact of RoB in individual studies on the results of the meta-analysis or other evidence synthesis?	Yes	Yes	Yes	N/a	N/a	Yes	No
*Q13: Did the review authors account for RoB in individual studies when interpreting/discussing the results of the review?	Yes	Yes	Yes	Yes	Yes	No	No
Q14: Did the review authors provide a satisfactory explanation for, and discussion of, any heterogeneity	Yes	Yes	Yes	Yes	No	No	Yes

observed in the results of the review?							
*Q15: If they performed quantitative synthesis did the review authors carry out an adequate investigation of publication bias and discuss its likely impact on the results of the review?	Yes	Yes	No	N/a	N/a	No	Yes
Q16: Did the review authors report any potential sources of conflict of interest, including any funding they received for conducting the review?	Yes	Yes	Yes	Yes	Yes	Yes	Yes
**Overall Rating**	Moderate	Moderate	Low	Moderate	Moderate	Critically low	Low

	**AMSTAR 2 Ratings**						
**AMSTAR 2 Questions**	Hovadick et al., 2021	Kishita et al., 2018	Knowles et al., 2021	Kustanti et al., 2021	Lee et al., 2020b	Lee et al., 2020a	Lee et al., 2021
Q1: Did the research questions and inclusion criteria for the review include the components of PICO?	Yes	Yes	Yes	Yes	Yes	Yes	Yes
*Q2: Did the report of the review contain an explicit statement that the review methods were established prior to the conduct of the review and did the report justify any significant deviations?	No	No	No	Yes	No	No	Yes
Q3: Did the review authors explain their selection of the study designs for inclusion in the review?	Yes	No	No	No	No	Yes	Yes
*Q4: Did the review authors use a comprehensive literature search strategy?	Partial yes	Partial yes	Partial yes	Yes	Partial yes	Partial yes	Partial yes
Q5: Did the review authors perform study selection in duplicate?	Yes	Yes	Yes	Yes	Yes	No	Yes
Q6: Did the review authors perform data extraction in duplicate?	Yes	Yes	Yes	No	No	No	Yes
Q7: Did the review authors provide a list of excluded studies and justify the exclusions?	Yes	No	No	No	Partial yes	No	No
Q8: Did the review authors describe the included studies in adequate detail?	Yes	Partial yes	Partial yes	No	Partial yes	Yes	Partial yes
*Q9: Did the review authors use a satisfactory technique for assessing the risk of bias (RoB) in individual studies that were included in the review?	Yes	No	Yes	Yes	Partial yes	Yes	Yes
Q10: Did the review authors report on the sources of funding for the studies included in the review?	Yes	No	No	No	No	No	No
*Q11: If meta-analysis was performed did the review	N/a	Yes	No	Yes	Yes	Yes	Yes

authors use appropriate methods for statistical combination of results?							
Q12: If meta-analysis was performed, did the review authors assess the potential impact of RoB in individual studies on the results of the meta-analysis or other evidence synthesis?	N/a	No	No	Yes	Yes	No	Yes
*Q13: Did the review authors account for RoB in individual studies when interpreting/discussing the results of the review?	Yes	No	No	Yes	Yes	No	Yes
Q14: Did the review authors provide a satisfactory explanation for, and discussion of, any heterogeneity observed in the results of the review?	No	Yes	Yes	Yes	Yes	Yes	Yes
*Q15: If they performed quantitative synthesis did the review authors carry out an adequate investigation of publication bias and discuss its likely impact on the results of the review?	N/a	No	No	Yes	No	Yes	No
Q16: Did the review authors report any potential sources of conflict of interest, including any funding they received for conducting the review?	Yes	Yes	Yes	No	Yes	Yes	Yes
**Overall Rating**	Low	Critically low	Critically low	Moderate	Critically low	Critically low	Low

	**AMSTAR 2 Ratings**						
**AMSTAR 2 Questions**	Liu et al., 2022	Lucero et al., 2019	Martensson et al., 2023	Martis et al., 2023	Moore et al., 2020	Mou et al., 2021	Sun et al., 2022a
Q1: Did the research questions and inclusion criteria for the review include the components of PICO?	Yes	Yes	Yes	Yes	Yes	Yes	Yes
*Q2: Did the report of the review contain an explicit statement that the review methods were established prior to the conduct of the review and did the report justify any significant deviations?	Partial yes	Yes	Yes	Yes	Partial yes	Yes	Yes
Q3: Did the review authors explain their selection of the study designs for inclusion in the review?	Yes	No	No	No	No	No	No
*Q4: Did the review authors use a comprehensive literature search strategy?	Partial yes	Yes	Partial yes	Partial yes	Partial yes	Partial yes	Partial yes
Q5: Did the review authors perform study selection in duplicate?	Yes	Yes	Yes	Yes	Yes	Yes	Yes
Q6: Did the review authors perform data extraction in	Yes	Yes	Yes	Yes	Yes	Yes	No

duplicate?							
Q7: Did the review authors provide a list of excluded studies and justify the exclusions?	No	No	No	No	Yes	No	No
Q8: Did the review authors describe the included studies in adequate detail?	Partial yes	Yes	Yes	Partial yes	Yes	Partial yes	Partial yes
*Q9: Did the review authors use a satisfactory technique for assessing the risk of bias (RoB) in individual studies that were included in the review?	Yes	Yes	Yes	Yes	Yes	Yes	Yes
Q10: Did the review authors report on the sources of funding for the studies included in the review?	No	No	Yes	No	No	No	No
*Q11: If meta-analysis was performed did the review authors use appropriate methods for statistical combination of results?	Yes	No	Yes	Yes	N/a	Yes	Yes
Q12: If meta-analysis was performed, did the review authors assess the potential impact of RoB in individual studies on the results of the meta-analysis or other evidence synthesis?	No	No	Yes	No	N/a	Yes	No
*Q13: Did the review authors account for RoB in individual studies when interpreting/discussing the results of the review?	Yes	Yes	Yes	Yes	Yes	Yes	Yes
Q14: Did the review authors provide a satisfactory explanation for, and discussion of, any heterogeneity observed in the results of the review?	Yes	No	Yes	Yes	Yes	Yes	Yes
*Q15: If they performed quantitative synthesis did the review authors carry out an adequate investigation of publication bias and discuss its likely impact on the results of the review?	Yes	No	Yes	Yes	N/a	No	Yes
Q16: Did the review authors report any potential sources of conflict of interest, including any funding they received for conducting the review?	No	Yes	Yes	Yes	Yes	Yes	Yes
**Overall Rating**	Moderate	Low	Moderate	Moderate	Moderate	Low	Moderate

	AMSTAR 2 Ratings						
**AMSTAR 2 Questions**	Sun et al., 2022b	Teahan et al., 2020	Wang et al., 2020	Wang et al., 2021	Wiegelmann et al., 2021	Williams et al., 2019	Zabihi et al., 2020
Q1: Did the research questions and inclusion criteria for the review include the components of PICO?	Yes	Yes	Yes	Yes	Yes	Yes	Yes
*Q2: Did the report of the review contain an explicit statement that the review methods were established prior to the conduct of the review and did the report justify any significant deviations?	Yes	No	Yes	Yes	No	No	Yes
Q3: Did the review authors explain their selection of the study designs for inclusion in the review?	No	No	No	No	Yes	Yes	No
*Q4: Did the review authors use a comprehensive literature search strategy?	Partial yes	Partial yes	Partial yes	Partial yes	Partial yes	Partial yes	Partial yes
Q5: Did the review authors perform study selection in duplicate?	Yes	Yes	Yes	Yes	Yes	No	Yes
Q6: Did the review authors perform data extraction in duplicate?	Yes	No	No	Yes	No	No	Yes
Q7: Did the review authors provide a list of excluded	No	No	No	No	No	No	Yes

studies and justify the exclusions?							
Q8: Did the review authors describe the included studies in adequate detail?	Partial yes	Yes	Partial yes	Partial yes	Partial yes	No	Partial yes
*Q9: Did the review authors use a satisfactory technique for assessing the risk of bias (RoB) in individual studies that were included in the review?	Yes	No	Yes	Yes	No	Yes	Yes
Q10: Did the review authors report on the sources of funding for the studies included in the review?	No	No	No	No	No	No	No
*Q11: If meta-analysis was performed did the review authors use appropriate methods for statistical combination of results?	Yes	Yes	Yes	N/a	N/a	Yes	Yes
Q12: If meta-analysis was performed, did the review authors assess the potential impact of RoB in individual studies on the results of the meta-analysis or other evidence synthesis?	Yes	No	Yes	N/a	N/a	Yes	Yes
*Q13: Did the review authors account for RoB in individual studies	Yes	No	Yes	Yes	No	Yes	Yes

when interpreting/discussing the results of the review?							
Q14: Did the review authors provide a satisfactory explanation for, and discussion of, any heterogeneity observed in the results of the review?	Yes	Yes	Yes	No	Yes	Yes	Yes
*Q15: If they performed quantitative synthesis did the review authors carry out an adequate investigation of publication bias and discuss its likely impact on the results of the review?	Yes	No	Yes	N/a	N/a	Yes	Yes
Q16: Did the review authors report any potential sources of conflict of interest, including any funding they received for conducting the review?	Yes	Yes	Yes	Yes	Yes	Yes	Yes
**Overall Rating**	Moderate	Critically low	Moderate	Moderate	Critically low	Low	Moderate

	AMSTAR 2 Ratings					
**AMSTAR 2 Questions**	Zhao et al., 2019	Zheng et al., 2021	Zhou et al., 2021	Zhu et al., 2021	Zulkifley et al., 2020	
Q1: Did the research questions and inclusion criteria for the review include the components of PICO?	Yes	Yes	Yes	Yes	Yes	
*Q2: Did the report of the review contain an explicit statement that the review methods were established prior to the conduct of the review and did the report justify any significant deviations?	No	Yes	Yes	No	Yes	
Q3: Did the review authors explain their selection of the study designs for inclusion in the review?	Yes	No	No	No	No	
*Q4: Did the review authors use a comprehensive literature search strategy?	Partial yes	Partial yes	Partial yes	Partial yes	Partial yes	
Q5: Did the review authors perform study selection in duplicate?	Yes	Yes	Yes	Yes	No	
Q6: Did the review authors perform data extraction in duplicate?	Yes	Yes	Yes	Yes	Yes	
Q7: Did the review authors provide a list of excluded studies and justify the exclusions?	No	No	No	No	No	
Q8: Did the review authors describe the included studies in adequate detail?	Partial yes	Partial yes	Yes	Partial yes	Partial yes	
*Q9: Did the review authors use a satisfactory technique for assessing the risk of bias (RoB) in individual studies that were included in the review?	Yes	Yes	Yes	Yes	No	
Q10: Did the review authors report on the sources of funding for the studies included in the review?	No	No	No	No	No	
*Q11: If meta-analysis was performed did the review	Yes	N/a	Yes	Yes	N/a	

authors use appropriate methods for statistical combination of results?						
Q12: If meta-analysis was performed, did the review authors assess the potential impact of RoB in individual studies on the results of the meta-analysis or other evidence synthesis?	No	N/a	Yes	No	N/a	
*Q13: Did the review authors account for RoB in individual studies when interpreting/discussing the results of the review?	Yes	Yes	Yes	Yes	No	
Q14: Did the review authors provide a satisfactory explanation for, and discussion of, any heterogeneity observed in the results of the review?	Yes	Yes	Yes	Yes	No	
*Q15: If they performed quantitative synthesis did the review authors carry out an adequate investigation of publication bias and discuss its likely impact on the results of the review?	Yes	N/a	Yes	Yes	N/a	
Q16: Did the review authors report any potential sources of conflict of interest, including any funding they received for conducting the review?	Yes	Yes	Yes	Yes	Yes	
**Overall Rating**	Low	Moderate	Moderate	Low	Critically low	

**As discussed in Shea et al., 2017 authors should decide on the elements most critical to assess given the PICO question of the study. The following critical questions were identified by the study team as essential to interpreting the overall AMSTAR 2 interpretation: 2, 4, 9, 11, 13 and 15.
